# Factors affecting the bacterial community composition and heterotrophic production of Columbia River estuarine turbidity maxima

**DOI:** 10.1002/mbo3.522

**Published:** 2017-08-06

**Authors:** Lydie Herfort, Byron C. Crump, Caroline S. Fortunato, Lee Ann McCue, Victoria Campbell, Holly M. Simon, António M. Baptista, Peter Zuber

**Affiliations:** ^1^ NSF Science & Technology Center for Coastal Margin Observation & Prediction (CMOP) Portland OR USA; ^2^ Institute of Environmental Health, Oregon Health & Science University Portland OR USA; ^3^ College of Earth, Ocean, and Atmospheric Sciences Oregon State University Corvallis OR USA; ^4^ Horn Point Laboratory University of Maryland Center for Environmental Science Cambridge MD USA; ^5^ Pacific Northwest National Laboratory Richland WA USA; ^6^ Present address: Josephine Bay Paul Center Marine Biological Laboratory Woods Hole MA USA; ^7^ Present address: Department of Medicine University of Washington Seattle WA USA

**Keywords:** Columbia River estuary, estuarine turbidity maxima, free‐living bacteria, particle‐attached bacteria

## Abstract

Estuarine turbidity maxima (ETM) function as hotspots of microbial activity and diversity in estuaries, yet, little is known about the temporal and spatial variability in ETM bacterial community composition. To determine which environmental factors affect ETM bacterial populations in the Columbia River estuary, we analyzed ETM bacterial community composition (Sanger sequencing and amplicon pyrosequencing of 16S rRNA gene) and bulk heterotrophic production (^3^H‐leucine incorporation rates). We collected water 20 times to cover five ETM events and obtained 42 samples characterized by different salinities, turbidities, seasons, coastal regimes (upwelling vs. downwelling), locations, and particle size. Spring and summer populations were distinct. All May samples had similar bacterial community composition despite having different salinities (1–24 PSU), but summer non‐ETM bacteria separated into marine, freshwater, and brackish assemblages. Summer ETM bacterial communities varied depending on coastal upwelling or downwelling conditions and on the sampling site location with respect to tidal intrusion during the previous neap tide. In contrast to ETM, whole (>0.2 μm) and free‐living (0.2–3 μm) assemblages of non‐ETM waters were similar to each other, indicating that particle‐attached (>3 μm) non‐ETM bacteria do not develop a distinct community. Brackish water type (ETM or non‐ETM) is thus a major factor affecting particle‐attached bacterial communities. Heterotrophic production was higher in particle‐attached than free‐living fractions in all brackish waters collected throughout the water column during the rise to decline of turbidity through an ETM event (i.e., ETM‐impacted waters). However, free‐living communities showed higher productivity prior to or after an ETM event (i.e., non‐ETM‐impacted waters). This study has thus found that Columbia River ETM bacterial communities vary based on seasons, salinity, sampling location, and particle size, with the existence of three particle types characterized by different bacterial communities in ETM, ETM‐impacted, and non‐ETM‐impacted brackish waters. Taxonomic analysis suggests that ETM key biological function is to remineralize organic matter.

## 1 INTRODUCTION

In the land‐ocean continuum, estuaries act as conduits for transport, transformation and production of organic carbon and nutrients. The fate of the substances transported to and produced within estuaries depends on the specific physical, chemical, and biological processes occurring in each estuarine ecosystem. Whether estuaries are predominantly autotrophic or heterotrophic systems (Gattuso, Frankignoulle, & Wollast, 1998; Maher & Eyre, 2012), their microbiota often play an important role in overall ecosystem functioning. Their microbial communities are influenced by the strong environmental gradients typically occurring in these systems, with associated changes in their community composition, activity, and lifestyles (Almeida, Cunha, & Alcântara, 2002; Karrascha, Ullrichb, Mehrensa, & Zimmermann‐Timm, 2003; Lapoussière, Michel, Starr, Gosselin, & Poulin, 2011; Santos et al., 2014). Hence, shifts in bacterial community composition along salinity gradients are well documented (Bouvier & del Giorgio, 2002; Campbell & Kirchman, 2013; Dupont et al., 2014; Fortunato, Herfort, Zuber, Baptista, & Crump, 2012; Herlemann, Lundin, Andersson, Labrenz, & Jürgens, 2016; Herlemann et al., 2011; Kirchman, Dittel, Malmstrom, & Cottrell, 2005; Liu et al., 2015; Ortega‐Retuerta, Joux, Jeffrey, & Ghiglione, 2013).

In dynamic estuaries, diverse microbial communities are formed during mixing of fresh and marine water masses. For example, in the fast‐flowing river‐dominated tidal Columbia River estuary (CRE) (2,000–15,000 m^3^ s^−1^, Baptista et al., 2015), the free‐living bacterial (0.2–3 μm) assemblage of a brackish water sample was observed to include a mixture of bacteria found in low and high salinity samples (Crump, Armbrust, & Baross, 1999). In contrast, the particle‐attached bacteria (>3 μm) of this brackish sample were phylogenetically distinct from those of the end‐member waters. Phylogenetic differences between particle‐attached and free‐living bacterial communities in coastal margins have been noted in numerous studies (DeLong, Franks, & Alldredge, 1993; Acinas, Antón, & Rodríguez‐Valera, 1999; Crump et al., 1999; Moesender, Winter, & Herndl, 2001; Ghiglione, Conan, & Pujo‐Pay, 2009; Smith, Zeigler‐Allen, Allen, Herfort, & Simon, 2013; Jackson, Millar, Payne, & Ochs, 2014; among others), but the opposite has also been observed (Ghiglione et al., 2007; Hollibaugh, Wong, & Murrell, 2000; Riemann & Winding, 2001). Similarly, while the bulk of bacterial activity in some systems is associated with particle‐attached rather than free‐living bacteria (Campbell & Kirchman, 2013; Crump & Baross, 1996; Ghiglione et al., 2007; Kirchman, 1993), this is not always the case (Karrascha et al., 2003; Painchaud & Therriault, 1989; Plummer, Owens, & Herbert, 1987). These discrepancies likely reflect the range of prevalent environmental forcings that exist between study sites (e.g., different carbon content of particles, Ortega‐Retuerta et al., 2013). For generating global conceptual models of how free‐living and particle‐attached bacteria respond to environmental change, it is essential that we gain further knowledge of the factors affecting these communities in specific environments. The paucity of knowledge applied to understand the environmental factors shaping particle‐attached and free‐living communities is likely exacerbated by the fact that in most aquatic microbial studies, the distinction between the two communities is often not considered or the particle‐attached fraction is simply omitted from analysis because of prefiltration (Rieck, Herlemann, Jürgens, & Grossart, 2015). Particle‐attached bacteria can represent up to 90% of total estuarine bacterial abundance (Lapoussière et al., 2011; Simon, Grossart, Schweitzer, & Ploug, 2002; Zimmermann, 1997) and in fast‐flowing estuaries, physical features and events that extend particle residence time are likely important for shaping the particle‐attached community.

Estuarine turbidity maxima (ETM) are such events/features that increase estuarine particle residence time. Typical of temperate well‐mixed tidal estuaries, ETM are transient sedimentary features generated during tidal reversals as the deep salinity intrusion interacts with residual bottom currents (Jay & Musiak, 1996; Sanford, Suttles, & Halka, 2001). In the Columbia River estuary, ETM turbidity varies (50 to >200 mg L^−1^) according to the season, diurnal tidal cycle, and spring/neap tidal cycle, with largest ETM usually observed during spring flood tides (Small & Morgan, 1994; Small & Prahl, 2004). Columbia River ETM acts as a three‐dimensional SPM conveyor belt, with accumulation of new bioactive materials during neap‐tide stratification, followed by erosion of bioactive materials that have been microbiologically transformed during spring‐tide mixing (Small & Prahl, 2004). This process also involves transport of SPM from shallows to main channels during ebb tide and from channels to shoals during flood tide (Lopez, Baptista, & Spitz, 2012). Thus, particles incorporated into ETM have multiple origins: river, ocean (including oxygen‐depleted waters during upwelling events), and estuarine sediment. Particle dynamics in the estuary are extremely complex (Sherwood, Creager, Roy, Gelfenbaum, & Dempsey, 1984). In a simplified view, the resuspended SPM in the ETM can be thought of as particles that are too big to be washload (particles that settle too slowly to remain in the estuary) and too small to be bedload (particles that settle rapidly and become part of the estuarine bed), and can thus be effectively trapped in an ETM (Fain, Jay, Wilson, Orton, & Baptista, 2001).

ETM are recognized to have a profound influence on estuarine biogeochemical and biological processes (Goosen, Kromkamp, Peene, van Rijswijk, & van Breugel, 1999; Herman & Heip, 1999; Jay & Musiak, 1996). A recent metagenome analysis of size‐fractionated Columbia River ETM water confirmed that ETM free‐living and particle‐attached bacterial assemblages were phylogenetically different from each other, with evidence for the existence of anoxic microzones in ETM particles (Smith et al., 2013). Crump et al. (1999) postulated that the free‐living ETM bacteria do not have opportunities to develop their own distinct community because, in contrast to particle‐attached ETM bacteria, they are rapidly flushed out of the estuary. ETM are also typically sites of enhanced microbial heterotrophic production (Goosen et al., 1999). In the CRE, ETM particle‐attached bacterial communities are more active (10–100 times) than their free‐living counterparts (based on bulk rates of ^3^H‐thymidine incorporation into >3 μm and 0.2–3 μm fractions) and are responsible for as much as 90% of the microbial secondary production measured in the estuarine water column (Crump & Baross, 1996; Crump, Baross, & Simenstad, 1998). Particle‐attached ETM bacteria also have a higher abundance of gene encoding products that function in phytoplankton decomposition, assimilation of diatom exopolysaccharide carbon, and utilization of dissolved organic carbon, more so than free‐living ETM bacteria (Smith et al., 2013).

The ETM bacteria are thus likely to be major contributors to the biogeochemical transformations ongoing in the Columbia River estuary. Our knowledge of the bacteria of ETM in this system is nonetheless limited by the fact that genetic studies conducted thus far have been carried out on only two ETM samples (Crump et al., 1999; Smith et al., 2013). This is particularly limiting given the high temporal and spatial dynamics of physical and biological processes in the CRE. ETM develop over a wide range of physical conditions (during four distinct physical regimes, over a wide salinity range, and in two channels influenced by different patterns of circulation), and overall estuarine bacterial assemblages vary seasonally and across the salinity gradient (Fortunato & Crump, 2011; Fortunato et al., 2012). Hence, it remains unclear how the bacterial assemblages (particle‐attached and free‐living) of ETM events vary spatially and temporally. In addition, it is not clear which environmental factors specifically affect these ETM communities.

Our goal was therefore, to improve our knowledge of the biological complexity associated with ETM in this fast‐flowing estuary by studying more ETM events than the two that have been previously characterized, so as to eventually be able to better constrain estuarine biogeochemical models. More specifically, this study, focused on five Columbia River ETM events (mostly water column samples), was undertaken to determine if variation in bacterial community composition (16S rRNA gene sequences) and bulk activity (^3^H‐leucine incorporation rates) is related to differences in seasonality (and associated co‐varying factors), salinity, ETM location between and within channels, and particle dynamics. We first studied bacterial communities of ETM events in different seasons (spring and summer) using Sanger sequencing. Then, we more specifically investigated the particle‐attached and free‐living bacterial communities of summer ETM events using pyrosequencing. To gather preliminary data on whether underlying channel sediment contributes as a major source of bacteria to the ETM, we also analyzed the surface sediment at an ETM site.

The data confirmed a strong influence of season and salinity on water column bacterial communities, although salinity history was what mattered for ETM. Our study also uncovered the existence of different particle‐attached bacterial communities in ETM, ETM‐impacted, and non‐ETM‐impacted brackish waters. Finally, based on our taxonomic composition data, ETM were found to be sites of potential organic matter remineralization. Together our findings highlight the complexity of ETM in a fast‐flowing estuary that will need to be implemented in future biogeochemical models.

## 2 MATERIALS AND METHODS

### 2.1 Sample acquisition

**Figure 1:**
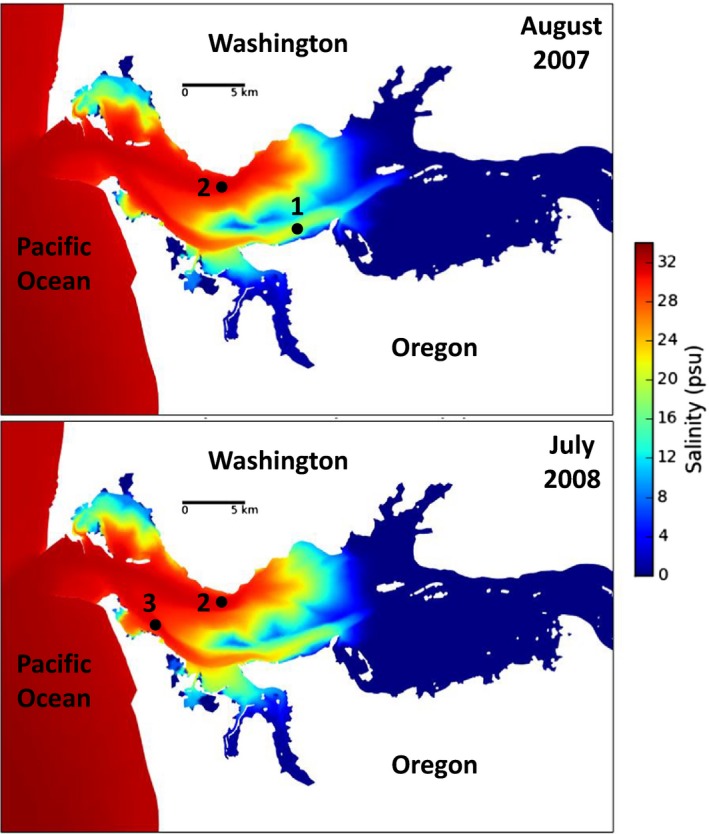
Maps of the CRE showing sampling sites (1–3) and corresponding simulations of maximum salinities of bottom water oceanic intrusions in August 2007 (top panel) and July 2008 (bottom panel)

Eulerian time series were conducted on board the R/V Barnes on 26–27 August 2007 and 18 and 20 July 2008 during spring flood tides to collect bottom water (1 m above estuarine bed) before, during, and after ETM events (Table 1) in North and South channels (Figure 1). An ETM event is described here as tidally generated rise, peak (ETM), and fall of turbidity in bottom waters. Several estuarine mechanisms are able to generate turbidity measurements above background sources, but we are confident the high turbidity samples collected for this study are associated with ETM because the turbidity concentrations described here were observed in the context of tidal phase, velocity, and salinity conditions that are consistent with the representation and location of the ETM in the sediment model of the Center for Coastal Margin Observation & Prediction (CMOP) (Lopez et al., 2012). Bottom water from an ETM event can be either ETM peak brackish water (henceforth referred to as ETM) or not peak ETM brackish waters (henceforth referred to as brackish non‐ETM). Surface water (1 m) was also obtained during 2007 ETM peaks (Table 1). A similar sampling scheme was carried out onboard the R/V New Horizon on 23 May 2009 in the North channel (Figure 1), resulting in the collection of an additional four water samples (Table 1). All water samples were collected using a high volume, low pressure, air‐driven pump attached to a cage equipped with a Seabird conductivity‐temperature‐depth (CTD) sensor. A suite of environmental sensors, including turbidity, oxygen, and fluorescence, was fitted onto the CTD cage. Water from the same CTD cast was employed for samples collected for Sanger sequencing, pyrosequencing, and biogeochemical analyses.

**Table 1:** Water and sediment sample descriptions and associated physical parameters

Sample description					Sample codes		Physical parameters of water			
Sample	Date	Estuary channel^a^	Phase of ETM development	Depth	Sanger	Pyro‐	Salinity	Temperature (°C)	SPM (mg L^−1^)	Estuary physical regime
					Sequencing					
Water	Aug‐07	South (site1)	Pre‐ETM	Bottom	A	A/Af	5.6	19.3	9	Partially mixed
			Starting ETM	Bottom	B	B/Bf	10.3	18.3	16	Partially mixed
			ETM peak	Surface	C	C	0.9	20.4	7	Partially mixed
			**ETM peak**	**Bottom**	**D**	**D/Df**	**12.1**	**17.7**	**85**	**Partially mixed**
			Decreasing ETM	Bottom	E	E/Ef	11.9	17.8	79	Partially mixed
			Post‐ETM	Bottom	F	F/Ff	13.1	17.4	19	Partially mixed
		North (site2)	Pre‐ETM	Bottom	G	G/Gf	9.0	18.2	20	Partially mixed
			**ETM peak**	**Bottom**	**H**	**H**/**Hf**	**12.3**	**17.4**	**49**	**Partially mixed**
			ETM peak	Surface		U/Uf	6.8	18.8	8	Partially mixed
			Post‐ETM	Bottom	I	I/If	29.5	12.2	17	Partially mixed
	Jul‐08	South (site3)	Pre‐ETM	Bottom	J	J	11.0	15.7	40	Partially mixed
			**ETM peak**	**Bottom**	**K**	**K**	**20.1**	**12.6**	**64**	**Partially mixed**
			Post‐ETM	Bottom		R	31.4	8.4	46	Partially mixed
			Post‐ETM	Bottom	L	L	31.9	8.2	50	Partially mixed
		North (site2)	Pre‐ETM	Bottom		S	5.1	17.4	33	Partially mixed
			**ETM peak**	**Bottom**		**T**	**11.0**	**15.5**	**93**	**Partially mixed**
	May‐09	North (site2)	Pre‐ETM	Bottom	M		1.2	13.5	61	Time‐dep. salt wedge
			**ETM peak**	**Bottom**	**N**		**12.8**	**11.7**	**169**	**Time‐dep. salt wedge**
			ETM peak	Surface	O		1.4	13.8	10	Time‐dep. salt wedge
			Post‐ETM	Bottom	P		24.1	9.7	56	Time‐dep. salt wedge
Sediment	Aug‐07	North (site2)	Post‐ETM		Q					Partially mixed

Samples A–T and Af–If represent the whole (>0.2 μm) and prefiltered (0.2–3 μm) water samples, respectively. Sample Q is a surface sediment sample collected after an ETM event. Samples collected from the bottom water during the peak of each ETM are highlighted in bold.

a Location of sites 1–3 is provided in Figure 1.

For all phylogenetic analyses of whole water (>0.2 μm fraction) collected during all three sampling campaigns, 1 L of water was filtered through 0.2 μm pore‐size Sterivex filters (PES, ESTAR, Millipore) using a peristaltic pump. Samples were also fractionated to analyze the free‐living bacterial community (0.2–3 μm) using a gentle fractionation approach (Crump et al., 1999). Briefly, sampled water in a 20‐L bucket was allowed to flow up through a 3.0 μm pore size, 142‐mm diameter polycarbonate membrane (Isopore, Millipore) fitted at the end of a floating plastic cylinder. Water (1 L) within the cylinder (<3 μm fraction) was then filtered through a 0.2 μm pore‐size Sterivex filter using a peristaltic pump to collect the 0.2–3 μm fraction. Sterivex filters were preserved with RNAlater (Ambion) for Sanger sequencing, or with the DNA extraction buffer (DEB, Fortunato & Crump, 2011) for pyrosequencing, before storage at −80°C. Filters were kept on dry ice for transportation from ship to onshore laboratory. With our fractionation scheme, the whole water (>0.2 μm) contains both particle‐attached and free‐living bacteria, while the smaller (0.2–3 μm) size fraction includes free‐living bacteria and small particles (henceforth referred to as free‐living fraction). We collected water 20 times to cover five ETM events and obtained a total of 42 water samples (Table 1). Surface sediment was also collected using a grab sampler in August 2007 after sampling the North channel post‐ETM water (Table 1). The top 1 cm (~20 ml) was transferred to a 50 ml Falcon tube before adding 3 ml of RNAlater and storing at −80°C.

### 2.2 Sanger sequencing of 16S rRNA gene clone libraries

DNA was extracted, PCR‐amplified with universal bacterial primers 907f (5′‐AAA CTC AAA GGA ATTGAC GGG‐3′) (Santegoeds, Ferdelman, Muyzer, & de Beer, 1998) and 1492r (5′‐GGT TAC CTT GTT ACG ACT T‐3′) (Lane, 1991), cloned, sequenced, analyzed, and quality‐controlled as described in Herfort et al. (2012). This generated 2191 sequences (81–194 sequences per sample) after removal of chloroplast sequences (Table S1 and Figure S1). Each sequence has a length of 395 bp as it was trimmed between positions 981–1,434 (including V4–V9 regions). These sequences have been deposited in the GenBank database under accession numbers KT449888–KT452057. Operational taxonomic units (OTUs) (based on 97% similarity) and rarefaction curves (Figure S1) were generated using MOTHUR (v. 1.34.1; Schloss et al., 2009). These rarefaction curves show that the bacterial communities were undersampled, thus indicating that our study is focused on abundant organisms, not the rare biosphere. Taxonomic affiliation of OTUs was determined using the Ribosomal Database Project Classifier tool (v. 10.x; Wang, Garrity, Tiedje, & Cole, 2007).

### 2.3 Amplicon pyrosequencing of 16S rRNA gene reads

DNA was extracted and purified as described by Fortunato and Crump (2011) and underwent further processing for amplicon pyrosequencing as documented in Fortunato et al. (2013). In short, 16S rRNA genes were PCR‐amplified using primers focusing on the V2 region, 27F with the 454B FLX linker (5′‐GCCTTGCCAGCCCGCTCAGTCAGRGTTTGATYMTGGCTCAG‐3′) and 338R with 454A linker and a unique 8‐bp barcode for each sample, denoted by N in primer sequence (5′‐GCCTCCCTCGCGCCATCAGNNNNNNNCATGCWGCCWCCCGTAGGWGT‐3′) (Hamady, Walker, Harris, Gold, & Knight, 2008). Pyrosequencing was performed on a Roche‐454 FLX pyrosequencer at the Engencore of the University of South Carolina (Columbia, SC, USA). As detailed in Fortunato et al. (2013), sequence data were quality‐controlled using AmpliconNoise and clustering into OTUs was done at the 97% similarity level using the QIIME (v. 1.8.0.) software package, employing pick_open_reference_otus.py with the usearch method (database: 97% sequence OTUs of the Silva v.111 16S dataset). Rarefaction curves (Figure S1) were generated in QIIME (v. 1.8.0.) software package. These rarefaction curves show that the bacterial communities were undersampled, thus indicating that our study is focused on abundant organisms, not the rare biosphere. Due to the resulting wide range in sequence numbers (149–718) after removal of chloroplast sequences, reads were randomly rarefied to 149 for all samples for further analysis (Table S1 and Figure S1). As was done in Crump, Amaral‐Zettler, and Kling (2012), to verify that this approach did not introduce bias, we also replicated all analyses based on relative abundances (OTU abundances normalized to sequence number in each individual sample) using the complete dataset of sequences. Results with the complete dataset showed similar broad patterns of community composition as those presented in this paper for the rarefied dataset (for details see Figure S2). This was confirmed by PROTEST analysis: *m* ^*2*^ **= 0.18, correlation = 0.90, *p *=* *.001. All pyrosequencing data used in this paper were part of a larger coastal margin sample set (Fortunato et al., 2013) and can be downloaded from the NCBI Sequence Read Archive database under accession number SRP006412.

### 2.4 Heterotrophic microbial production

Whole or prefiltered (<3 μm fraction) water was used to measure rates of heterotrophic microbial production for whole water and free‐living communities, respectively, as described in Herfort et al. (2012). Briefly, rates of ^3^H‐leucine (20 nmol/L final concentration at 69 Ci mmol^−1^, Amersham) incorporation into the cold trichloroacetic acid insoluble fraction were measured in a scintillation counter in four 1.7 ml subsamples incubated on a rotator at in situ temperatures for 1 hr. Rates of ^3^H‐leucine uptake were converted to rates of carbon production, assuming a conversion factor of 3.09 kg C mol leu^−1^ (Kirchman, Keil, Simon, & Welschmeyer, 1993).

### 2.5 Environmental variables

Samples were collected for gravimetric measurements of SPM concentrations by filtering water until clogging onto a preweighted polycarbonate membrane (1.0 μm pore size, Poretics) on the 2007 cruise (80–300 ml filtered), or glass fiber filter (GF/F, 0.8 μm pore size, Whatman) on the 2008–2009 cruises (114–200 ml filtered), before storage at −20°C. After oven‐drying each filter at ~50°C, the mass of SPM was determined to compute concentration using the recorded volume filtered (Sullivan, Prahl, Small, & Covert, 2001).

Other environmental data (Tables S2 and S4) were collected and analyzed as described in Herfort, Peterson, McCue, and Zuber (2011). Note that salinity, temperature, SPM, chlorophyll *a*, particulate organic carbon (POC), phosphate and nitrate values for samples A–F have already been published in Herfort et al. (2011). Data on Columbia River discharge at Bonneville Dam (Oregon, USA), water elevation at Astoria (NOAA station 9439040), and North‐South wind speed (NOAA National Data Buoy Center station 46029) were obtained from the CMOP website (http://www.stccmop.org/datamart/observation_network/dataexplorer).

### 2.6 Analysis of relationship between bacterial community composition and environmental variables

Sanger and pyrosequencing datasets gave comparable results as ordinations for the samples they have in common showed good concordance (PROTEST, *m* ^*2*^ = 0.39, correlation = 0.78, *p *= .001). Given that (1) these datasets were obtained from Sterivex filters that were fixed and extracted differently and (2) they both include additional samples not present in the other dataset, we, however, performed all analyses independently for each dataset.

For both Sanger and pyrosequencing datasets, OTU‐based bacterial community composition was represented by dendrograms of hierarchical clusters and by ordination plots. The Plymouth Routines In Multivariate Ecological Research (PRIMER) software v.6 (PRIMER‐E Ltd, UK) was used to generate these dendrograms based on a resemblance matrix of Bray–Curtis similarities computed from the square root transformation of the relative abundance of the OTUs (with whole and rarefied dataset being used for Sanger and pyrosequencing, respectively). To test for evidence of significant internal structure, similarity profile analysis (SIMPROF; Clarke, Somerfield, & Gorley, 2008) was also performed in PRIMER. These same resemblance matrices were also used to run a Principal Coordinate Analysis (PCoA) in the PERMANOVA+ add‐on package of PRIMER v.7. To further identify the OTU that best correlated with these patterns, we performed correlation analyses (Pearson Product‐Moment Correlation Coefficient) between OTUs and patterns using the CORREL function in EXCEL (assigning 1 or 0 to samples to defined patterns, e.g., 1 for summer and 0 for spring).

The relationship between environmental data and bacterial OTUs diversity and abundance was first examined using BEST‐BIOENV in PRIMER v.6. This nonparametric analytical tool performs Spearman rank correlation coefficient analyses to determine the degree of association between the Bray–Curtis similarity matrix of the bacterial community composition and (normalized) environmental data. The BEST‐BIOENV analysis determines which subset of environmental data best explains the variability and generates a maximum coefficient of one if all variability is explained. One needs to keep in mind that it might be difficult to deconvolute a single most important factor because of the relatively small number of samples compared to environmental variables. So, based on the BEST result, we further compared a subset of environmental data using the nonparametric Mann–Whitney–Wilcoxon test in R software package (v. 3.2.2). The effect of various factors on bacterial communities of our Sanger and pyrosequencing datasets was then further examined by permutational analysis of variance (PERMANOVA) in PRIMER v.7 add‐on package, with default settings for the main test (unrestricted permutation of raw data and partitioning based on type III calculation of sum of squares) and 9,999 permutations. PERMANOVA also uses the Bray–Curtis dissimilarities matrix computed from the square root transformation of the relative abundance of OTUs. This is a permutation method, which unlike the BEST‐BIOENV routine is not a rank‐order match, but instead directly analyzes a resemblance matrix variance in response to a selected factor (e.g., season, water mass type). Several ordinations were compared using a Procrustean randomization test (PROTEST) (Gower, 1975; Jackson, 1995) using the Vegan (v. 2.4‐3) software package of R (v. 3.4.0).

### 2.7 Numerical simulations

The Virtual Columbia River, an in silico representation of the circulation and ecosystem dynamics in the extended estuary based on high‐resolution numerical models (Baptista et al., 2015), was used to generate maps of maximum salinities of bottom water oceanic intrusions using the DB31 simulation database (http://www.stccmop.org/datamart/virtualcolumbiariver/simulationdatabases/climatologicalatlas_db31). Estuarine regimes were determined for each sampling period as described in Geyer and MacCready (2014) using simulation data for a transect at the interdisciplinary endurance station SATURN‐03 located ~14 km upstream of the estuary entrance in the South channel.

## 3 RESULTS

### 3.1 Environmental variables associated with collected samples

Higher river discharges were recorded in spring (9,500 m^3^ s^−1^) compared to summer (3,300 and 4,500 m^3^ s^−1^ for 2007 and 2008) (Table S2). This difference was large enough to lead to distinct estuarine regimes (Table 1 and Figure S3). Although summer sampling periods had the same estuarine regime (partially mixed), they were characterized by different coastal physical regimes. The 2008 sampling was preceded by 9 days of upwelling conditions (defined as at least 3 days of South‐blowing winds), while downwelling conditions prevailed immediately before 2007 sampling (Figure S5).

Collected water had a wide range of salinities (0.9–31.9 PSU), temperatures (8.2–20.4°C), SPM (8–169 mg L^−1^), and dissolved oxygen (2.6–9.9 mg L^−1^) concentrations (Table 1 and S2). May 2009 ETM had a larger particle load (169 mg L^−1^) than those of August 2007 and July 2008 (49–93 mg L^−1^) (Table 1). This seasonal difference is not a reflection of the different filter employed since the same filter type was used for 2009 and 2008 samples. In fact, when comparing 2009 and 2008 waters (i.e., includes ETM) the SPM concentrations were statistically different (Mann–Whitney–Wilcoxon test *p *=* *.047, *n* = 10). In addition, seasonality in SPM concentration of ETM related to river flow has previously been reported (Fain et al., 2001; Small & Morgan, 1994). The two July 2008 oceanic samples (R and L) had a dissolved oxygen level below 2.7 mg L^−1^ and were thus qualified as having a mild level of O_2_ stress (Herfort et al., 2016).

### 3.2 Bacterial community composition based on Sanger sequencing data in relationship with environmental variables

**Figure 2:**
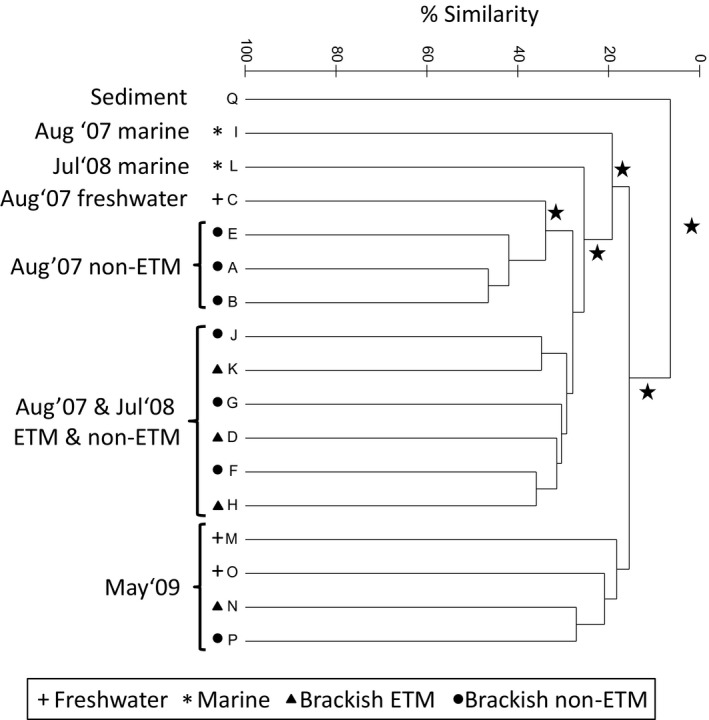
Dendrogram showing results of hierarchical cluster analysis of bacterial community composition based on Bray–Curtis similarities of the relative abundances of OTUs identified by Sanger sequencing of PCR‐amplified 16S rRNA genes from whole water (A–P) and surface sediment (Q) samples from the CRE. Significant divisions based on similarity profile analysis (SIMPROF) are highlighted by a star symbol

The largest difference in our Sanger sequencing dataset was observed between the North channel ETM‐site surface sediment (below the fluff layer) and all water samples (Figure 2). At the OTU level, the bacterial community of this surface sediment was distinct from that of water samples (Figure 2), and was characterized by a low proportion of Bacteroidetes and a high proportion of Gammaproteobacteria (29%, mostly Pseudomonadales) sequences (Table S3). Fifty‐seven percent of the sediment sequences were not found in the water dataset (Figure S4).

Sanger sequencing‐derived 16S rDNA OTUs of water samples were partitioned based on year of sampling, with May 2009 samples being significantly different (based on SIMPROF analysis) from July 2008 and August 2007 samples (Figure 2). The fact that summer samples do not also separate by year (PERMANOVA analysis of July 2008 vs. August 2007 communities: *df* = 1, pseudo‐*F* = 1.36, *p *=* *.073) indicates the dominance of seasonal differences (spring vs. summer). This bacterial seasonality has been previously established in large‐scale studies focused on the Columbia river‐to‐ocean gradient (Fortunato & Crump, 2015; Fortunato et al., 2012) and was confirmed for our ETM‐focused study by PERMANOVA analysis (*df* = 1, pseudo‐*F* = 2.59, *p *=* *.007). Bacteroidetes were the dominant class in the summer bacterial community (30% of all sequences, Table S3), with the majority being Flavobacteria (60% of Bacteroidetes, data not shown). In the spring, Bacteroidetes (24%) were also the second most abundant class of bacteria community following Gammaproteobacteria (30%) (Table S3). Spring was best correlated with three OTUs, two Flavobacteriaceae and one Bacteroidetes_incertae_sedis (Pearson coefficient: 0.94, 0.78, 0.76, respectively), while the best correlation with summer samples was only of 0.56 for an Oceanospirillaceae. When looking at brackish summer samples only, the top two bacteria associated (0.94) with peak ETM waters were a Prolixibacter and Micrococcineae, while Ilumatobacter was weakly correlated (0.65) with non‐ETM waters.

Many environmental parameters varied seasonally (e.g., increased river flow and reduced oceanic intrusion into the estuary during the spring freshet). To determine which of the measured environmental variables (Table S2) best explained the observed pattern in bacterial OTU diversity and abundance, BEST‐BIOENV analysis (based on Spearman's rank correlation coefficient analyses) was performed. The highest BEST‐BIOENV correlation (0.802) between Sanger sequencing‐derived OTUs of water samples and environmental variables (Table S2) was obtained with river discharge, salinity, and season. The next best correlation (0.801) added chlorophyll fluorescence to this list. When heterotrophic production values were added to the list of variables and sample O was removed (because only complete datasets can be analyzed), a correlation of 0.832 was found with salinity, season, and heterotrophic production. Chlorophyll fluorescence was larger in spring (May 2009: 5.1V) than summer (July 2008 and August 2009: 1.3V) (Table S2; Mann–Whitney–Wilcoxon test *p *=* *.0075, *n* = 16). The high chl *a* concentrations (determined by high‐performance liquid chromatographic) and ratios of chl *a* to particulate organic carbon (Table S4) point to a large, healthy May 2009 river (freshwater) phytoplankton bloom.

Salinity values did not vary with season (May 2009 vs. July 2008 and August 2007) in our dataset (Table 1, Mann–Whitney–Wilcoxon test: *p *=* *.68, *n* = 16), suggesting that salinity is by itself a structuring factor of the community. Even though May 2009 samples had widely different salinities (1–24 PSU), the OTU similarity profile analysis did not show a difference among the May 2009 communities (all four samples grouped together) (Figure 2). In contrast, the impact of salinity was apparent in the summer, when the bacterial compositions of freshwater and marine samples were distinct, and none of the samples of intermediate salinity grouped with those of the end‐member water masses (Figure 2). This was also shown on a PCoA plot (Figure 4a) where whole water bacterial communities separated on the vertical axis, with upwelled coastal water and freshwater marking end‐member extremes.

### 3.3 Bacterial community composition based in pyrosequencing data in relationship with environmental variables

Bacteroidetes were also the dominant class in the pyrosequencing dataset (35% of all sequences) (Table S5). When looking specifically at brackish summer samples, the top OTUs associated with peak ETM whole waters were a Rhodobacteraceae, a Gammaproteobacteria (no further taxonomic resolution), a Desulfobacteraceae, a Saprospiraceae, and two Flavobacteriaceae (Pearson coefficient: 0.84, 0.78, 0.75, 0.72, 0.71, 0.70, respectively). Other brackish summer samples (non‐ETM whole and prefiltered, and ETM prefiltered) were only weakly correlated (0.66) with a Pelagibacteraceae.

**Figure 3:**
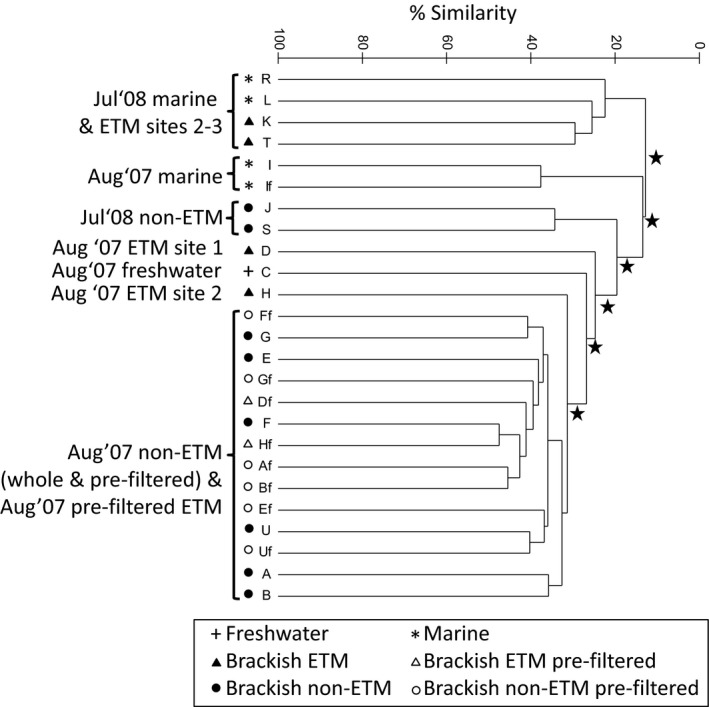
Dendrogram showing results of hierarchical cluster analysis of bacterial community composition based on Bray–Curtis similarities of the relative abundances of OTUs identified by pyrosequencing of PCR‐amplified 16S rRNA genes for whole (>0.2 μm; A–T) and prefiltered (0.2–3 μm; Af–If) water samples from the CRE. Significant divisions based on similarity profile analysis (SIMPROF) are highlighted by a star symbol

**Figure 4:**
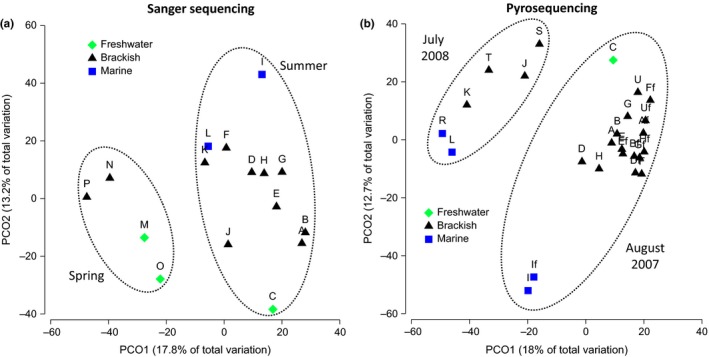
Principal coordinate analysis (PCoA) plots of bacterial community composition based on Bray‐Curtis similarities of the relative abundances of OTUs in water samples of the CRE. Two types of water sample were analyzed: whole (>0.2 μm; labeled A–T) and prefiltered (0.2–3 μm; labeled Af‐If). (a) Sanger sequencing spring and summer data showing separation of bacterial communities primarily based on season (May vs. July–August) and water mass (marine, brackish, freshwater). (b) Pyrosequencing of summer data showing separation of bacterial communities primarily based on summer sampling periods (July vs. August) and water mass salinity (marine, brackish, freshwater)

When comparing summer whole water samples, bacterial assemblages of brackish non‐ETM samples (i.e., brackish but not peak ETM bottom waters) did not cluster with those of end‐member waters (Figure 3), and were not further separated according to their salinity (Table 1). PERMANOVA analysis confirmed that summer estuarine bacterial communities of non‐ETM waters group into three distinct categories: marine, freshwater, and brackish (*df* = 2, pseudo‐*F* = 2.13, *p* = .001). This was also shown clearly on a PCoA plot (Figure 4b) where 2007 water samples separated on the vertical axis based on water masses, with again upwelled coastal water and freshwater marking the end‐member extremes. Similarly, salinity was one of the environmental variables that best explained the pyrosequencing data‐based bacterial community composition. The highest BEST‐BIOENV correlation (0.871) between OTU relative abundance and environmental variables (Table S2) was obtained with salinity, temperature, dissolved oxygen, heterotrophic production, and river discharge. When considering only the whole water samples of the pyrosequencing dataset, the highest BEST‐BIOENV correlation (0.858) was found with salinity and river discharge. When only prefiltered samples were analyzed, the highest correlation (0.710) was with dissolved oxygen, SPM, phosphate, and heterotrophic production. Interestingly, brackish non‐ETM whole water samples clustered with their free‐living counterparts and with the free‐living bacterial assemblages of ETM samples (Figure 3). PROTEST analysis of whole versus prefiltered waters showed good concordance between the datasets (*m* ^*2*^ = 0.63, correlation = 0.61, *p *=* *.005) likely because these only differ for ETM samples. July 2008 North and South channel ETM samples clustered with marine samples rather than with non‐ETM samples (Figure 3), suggesting a marine influence on these ETM bacterial communities. In contrast, in August 2007 North and South channel ETM and marine samples did not cluster together (Figure 3). The difference in oceanic influence on these ETM assemblages might be attributed to the different coastal physical regimes prevailing before these sampling periods, with downwelling in 2007 compared to upwelling conditions in 2008 (Figure S5). This distinction between summer sampling periods can be observed on a PCoA plot (Figure 4b), with horizontal separation between 2007 and 2008 bacterial communities. The different results obtained between our two sequencing approaches regarding separation of summer bacterial communities is likely due to the fact that to center our pyrosequencing analysis on different summer regimes (upwelling vs. downwelling conditions) additional July 2008 samples were analyzed.

When brackish and oceanic samples were compared, the later was correlated (Pearson coefficient: 0.90 and 0.78) with two OTUs related to the SUP05 cluster, which is an order of the Oceanospirillales. Given that those are microorganisms typically associated with oxygen minimum zones (Williams et al., 2012), to further explore the impact of coastal upwelling on ETM bacteria composition, we specifically looked at the abundance of microorganisms typically associated with oxygen‐depleted waters (Table 2). Note that these taxa were selected based on their enrichment in the metagenome of a Columbia River estuary ETM sample (Smith et al., 2013). Sequences related to the SUP05 cluster were highly represented in marine waters in 2007 (5.4%) and 2008 (4.7 and 9.4%), and were only detected in small amounts in one ETM sample (0.7%) in 2008 (Table 2). The abundance of sequences related to strict and facultative anaerobic bacteria in marine and ETM waters were statistically different between 2008 (8.2% ± 1.4%) and 2007 (2.5% ± 1.5) (Mann–Whitney–Wilcoxon test *p *=* *.047, *n* = 7) (Table 2). At first glance, low proportions of these sequences seemed to be associated with non‐ETM waters in 2008 (1.3%–2.0%; Table 3), but when corrected for SPM concentrations, their abundances follow a conservative mixing line for salinity (Figure S6), suggesting that those microorganisms from upwelled waters are transported into the estuary with particles and accumulate in ETM.

**Table 2:** Relative contribution in our pyrosequencing dataset of 16S rRNA gene sequences from bacteria typically associated with oxygen‐depleted waters in freshwater, brackish, and marine waters

	Aug‐07										Jul‐08					
	Non‐ETM								ETM		Non‐ETM				ETM	
Salinity	0.9	5.6	6.8	9.0	10.3	11.9	13.1	29.5	12.1	12.3	5.1	11.0	31.4	31.9	11	20.1
Sample code	C	A	U	G	B	E	F	I	D	H	S	J	L	R	T	K
SUP05	0.0	0.0	0.0	0.0	0.7	0.0	0.0	5.4	0.0	0.0	0.0	0.0	9.4	4.7	0.7	0.0
Desulfobacteraceae	0.0	0.0	0.0	0.0	1.3	0.7	0.0	0.7	1.3	1.3	0.7	0.7	2.0	2.7	4.7	2.7
Desulfobulbaceae	0.0	0.0	0.0	0.0	2.0	0.0	0.0	0.0	0.0	1.3	0.0	0.7	2.0	2.7	0.0	0.0
Desulfuromonadaceae	0.0	0.7	0.0	0.0	0.0	0.7	0.0	0.0	2.0	0.0	0.0	0.7	2.0	2.0	1.3	3.4
Anaerolineae	0.0	0.0	0.0	1.3	0.7	0.7	0.0	0.0	0.0	0.7	0.7	0.0	3.4	2.0	1.3	0.7

These taxa were selected based on their enrichment in the metagenome of a Columbia River estuary ETM sample (Smith et al., 2013) and represent class (Anaerolineae), order (SUP05), and family (Desulfobacteraceae, Desulfobulbaceae, Desulfuromonadaceae) taxonomic levels.

**Table 3:** Heterotrophic microbial production determined from bulk ^3^H‐leucine incorporation rates for whole (A–T) and prefiltered (Af–If) water samples

Sample description					Microbial secondary production (mg C L^−1^ h^−1^)		
Date	Channel^a^	Phase of ETM development	Depth	Code	Whole water >0.2 mm	Prefiltered water 0.2–3 mm	% of activity in particles
Aug‐07	South (site 1)	Pre‐ETM	Bottom	A/Af	0.5	0.26	48
		Starting ETM	Bottom	B/Bf	0.59	0.14	76
		ETM peak	Surface	C/Cf	0.24	0.08	67
		**ETM peak**	**Bottom**	**D**/**Df**	**1.42**	**0.26**	**82**
		Decreasing ETM	Bottom	E/Ef	0.87	0.13	85
		Post‐ETM	Bottom	F/Ff	0.67	0.55	18
	North (site 2)	Pre‐ETM	Bottom	G/Gf	1.17	0.67	43
		**ETM peak**	**Bottom**	**H**/**Hf**	**1.52**	**0.08**	**95**
		ETM peak	Surface	U/Uf	0.83	0.18	78
		Post‐ETM	Bottom	I/If	0.41	0.28	32
Jul‐08	South (site 3)	Pre‐ETM	Bottom	J	1.40	n.d.	n.d.
		**ETM peak**	**Bottom**	**K**	**1.66**	n.d.	n.d.
		Post‐ETM	Bottom	R	1.19	n.d.	n.d.
		Post‐ETM	Bottom	L	0.51	n.d.	n.d.
	North (site 2)	Pre‐ETM	Bottom	S	1.03	n.d.	n.d.
		**ETM peak**	**Bottom**	**T**	**2.49**	n.d.	n.d.
May‐09	North (site 2)	Pre‐ETM	Bottom	M	2.43	n.d.	n.d.
		**ETM peak**	**Bottom**	**N**	**4.76**	n.d.	n.d.
		ETM peak	Surface	O	n.d.	n.d.	n.d.
		Post‐ETM	Bottom	P	4.33	n.d.	n.d.

n.d., not determined. Samples collected from the bottom water during the peak of each ETM event are highlighted in bold.

a Location of sites 1–3 is provided in Figure 1.

Finally, the whole water bacterial assemblages of ETM were distinct in 2007, but grouped together in 2008 (Figure 3). This difference may be related to the location of the sampling site, and more precisely whether or not highly saline water masses reach these sites. The North channel sampling site (Figure 1) was at the same location in both years, and was subjected to large oceanic influences, as illustrated by the high salinity of the post‐ETM sample (29.5 PSU, Table 1). In contrast, South channel ETM locations were 15 km apart (Figure 1). Site 3, sampled in 2008, is located relatively close to the mouth of the estuary (river kilometer ~12) and thus experiences large oceanic influences, with up to 31.9 PSU measured in post‐ETM water (Table 1). Site 1, sampled in 2007, is situated more inland (river kilometer ~25) and it is therefore exposed to lower salinity water, with post‐ETM water of only 13.1 PSU (Table 1). Maps showing sampling locations in relation to the maximum salinities of bottom water during oceanic intrusion (Figure 1) help illustrate these differences between South channel sites as they clearly show maximum salinity of ~20 PSU in August 2007 at site 1 and ~30 PSU in July 2008 at site 3. Data from model simulations also show a maximum salinity of ~30 PSU for both months at site 2 in the North channel (Figure 1). These patterns of salinity intrusion are also supported by bottom water data from CMOP endurance monitoring stations that are located near our sampling sites (SATURN‐01, ‐03, and ‐04 for sites 2, 3, and 1; http://www.stccmop.org/datamart/observation_network). In July–August 2014, maximum salinities as high as 31 PSU were measured at SATURN‐01 and ‐03, but a maximum of only 22 PSU (with a daily peak often <16 PSU) was observed at SATURN‐04.

### 3.4 Microbial heterotrophic production

Microbial heterotrophic production rate in whole water samples was on average almost four times higher in spring compared to summer (3.84 and 1.04 mg C L^−1^ hr^−1^, respectively) (Table 3). For each time series, this rate was also highest in peak ETM bottom waters (Table 3).

To determine if the microbial activity associated with free‐living and particle‐attached bacteria fractions varies during the development of an ETM event, bulk rates of microbial heterotrophic production were measured on both whole and prefiltered water samples in August 2007. In both ETM and non‐ETM waters impacted by an ETM event—whether sampled during initiation of ETM formation or ETM decline, or at the surface during peak ETM—the majority (67%–95%) of this production was in the particulate fraction (Table 3). In contrast, for pre‐ and post‐ETM event waters (i.e., water not impacted by a turbidity event) most of the heterotrophic production was associated with the free‐living fraction (52%–82%; Table 3).

## 4 DISCUSSION

As dynamic mixing zones of oceanic and freshwater masses, estuaries and river plumes are often characterized by steep spatial and temporal gradients of physical, biogeochemical, and biological parameters. In turns, these gradients have the potential to impact the local bacterial community. Salinity has been shown to be an important environmental factor structuring bacterial communities in coastal margins (Bouvier & del Giorgio, 2002; Campbell & Kirchman, 2013; Dupont et al., 2014; Herlemann et al., 2011, 2016; Kirchman et al., 2005; Liu et al., 2015; Ortega‐Retuerta et al., 2013), including over the Columbia River‐to‐Pacific Ocean gradient (Fortunato & Crump, 2015; Fortunato et al., 2012). In our study, which comprises water samples collected exclusively within the CRE (0.9–31.9 PSU, Table 1), salinity was one of the factors that best determined overall bacterial community composition. In fact, non‐ETM summer estuarine bacterial assemblages clearly fell into three distinct categories: marine (>29 PSU), freshwater (1 PSU) or brackish (6–13 PSU) (Table 1 and Figure 4). This finding fits well with recent discovery of distinct brackish bacterial communities in the Baltic Sea, Chesapeake Bay, and Delaware Bay (Herlemann et al., 2011; Hugerth et al., 2015).

The effect of salinity on ETM bacterial assemblages was more subtle, but was detected when comparing ETM communities of North and South channels. ETM location is primarily determined by tidal asymmetry (i.e., the inequality in magnitude and duration in ebb and flood tidal currents) (Jay & Smith, 1990), with turbidity events emerging before or lagging behind the salt wedge. Sampling locations relative to the river mouth, and more precisely whether or not highly saline water masses reach these sites, were important factors that explained some of the variation in ETM bacterial community composition. Whole water bacterial assemblages of North and South channel ETM were similar when their locations near the mouth of the estuary exposed them to similarly high maximum salinity intrusions (Figures 1 and 3, and Table 1). In contrast, they were distinct when the sampling sites were differentially influenced by oceanic water masses (Figures 1 and 3, and Table 1). Therefore, salinity, as it relates to oceanic inputs, clearly impacts ETM bacterial communities.

In contrast, August 2007 ETM bacterial communities differed even though the samples had comparable salinities, while the community composition was similar for those of July 2008 despite a ~9 PSU difference in salinity (Figure 3 & Table 1). Differences between these four samples suggest that spring‐tide ETM bacterial communities are shaped mostly by local resuspension of SPM accumulated during the previous neap tide rather than by seaward advection that generates these events. This is consistent with SPM dynamics showing predominance of advective processes rather than local resuspension during neap tides, with lesser importance of advection during spring tides (Fain et al., 2001). An example of the local resuspension processes was provided when examining the abundance in ETM of strict and facultative anaerobic bacteria previously detected in the CRE water column in ETM and non‐ETM waters and in an oxygen‐depleted (0.7 mg L^−1^) deep (1,200 m) water sample from the Oregon coast (Smith et al., 2010, 2013). Despite their ~9 PSU difference in salinity, July 2008 ETM had a large abundance of these bacteria (Table 2), which likely originated from intrusion of low dissolved oxygen coastal waters derived from upwelling at the time of sampling and during the preceding neap tide (Figure S5). When corrected for SPM concentrations, their abundances in all samples (ETM and non‐ETM) followed a conservative mixing line for salinity (Figure S6). This suggests that those microorganisms originating from upwelled waters are transported into the estuary with particles and accumulate in ETM. In contrast, very few of the anaerobic bacterial sequences were detected in August 2007 (Table 2), a period dominated by coastal downwelling (Figure S5). The impact of exposure of salinity intrusion on ETM bacterial community composition is therefore especially significant during neap tides when particles accumulate.

Seasonality, and associated co‐varying factors, also had a strong influence, with distinct spring and summer bacterial communities and levels of bulk microbial heterotrophic production for both ETM and non‐ETM waters (Figure 2 and Table 3). Interestingly, May 2009 samples grouped together in our clustering analysis of bacterial community composition despite having widely different salinities (1–24 PSU) (Figure 2). This suggests that spring‐associated forcing overwhelms high salinity water impacts on the estuarine population during this period of higher river flow (Table S2). Three Bacteroidetes OTUs (Flavobacteriaceae and Bacteroidetes_incertae_sedis) were correlated with the spring season. This is good in agreement with the fact that Bacteroidetes were found to be involved in water column particle colonization and phytoplankton bloom degradation in the CRE (Smith, Herfort, Fortunato, Crump, & Simon, 2017). All spring samples also had high levels of bulk heterotrophic production than those collected in the summer (Table 3). These findings demonstrate that the large inputs of fresh organic matter to the estuary from riverine spring blooms (Table S4 and Lara‐Lara, Frey, & Small, 1990; Small et al., 1990; Prahl, Small, & Eversmeyer, 1997; Sullivan et al., 2001; Herfort et al., 2011) impact both bulk heterotrophic production and bacterial community composition.

Our data also highlighted the importance of particles in shaping the ETM bacterial community, but likely not particles from the underlying bedload sediment. Most (99%) of the sediment in the main channels is fine sand of 125–250 μm that is considered bedload, that is, particles that are deemed too large and fast settling to be resuspended during ETM events (Sherwood et al., 1984). Accordingly, the largest phylogenetic difference in our Sanger sequencing dataset was observed between our North channel bedload‐surface sediment sample (below the fluff layer) and all water samples (Figure 2). Admittedly this is only one sediment site (which is likely affected by the sand waves commonly occurring in this system) and we do not expect it to represent sediments of the entire estuary. Nonetheless, since this sample was collected at an ETM site after a resuspension event, the observed lack of community composition similarity between sediment and ETM does suggest that the underlying bedload‐surface sediment is not a major contributor of bacteria to the ETM. In contrast, results from recent studies indicated that lateral bay sediments are a likely source of particle‐attached bacteria to the ETM (Smith et al., 2015). Although this constitutes a first reporting of the idea that the bedload sediment in the main channel might contribute little as a source of bacteria to the ETM, sediment elsewhere (e.g., lateral bays and shoals) might be important for “seeding” the ETM with bacteria, and future studies should investigate this further.

In addition to identifying factors impacting the ETM and non‐ETM estuarine bacterial communities in whole water (>0.2 μm), one of our goals was also to examine the particle‐attached and free‐living bacteria of these two water types. This is especially important because pioneering phylogenetic studies that uncovered the distinction between free‐living and particle‐attached bacteria in the CRE were focused on the ETM, with no comparison with estuarine brackish non‐ETM waters (Crump et al., 1999; Smith et al., 2013). Our phylogenetic clustering analysis showed that the non‐ETM particle‐attached bacteria do not develop distinct estuarine communities (i.e., whole water [>0.2 μm] and free‐living [0.2–3 μm] assemblages were phylogenetically similar, Figure 3). This is likely, as originally introduced by Crump et al. (1999) when discussing free‐living ETM bacteria, because these non‐ETM particle‐attached bacteria do not benefit from the extended residence time of particulate material characteristic of ETM. In contrast, ETM free‐living and whole water (free‐living and particle‐attached) assemblages were different from one another, and in fact, the former was more similar to free‐living or whole water non‐ETM samples (Figure 3). Interestingly, taxonomic analysis reveals that a potential key function of ETM might be organic matter remineralization. Sulfate‐reducing bacteria (Desulfobacteraceae), Micrococcineae, and Bacteroidetes (Saprospiraceae and Flavobacteriaceae) were indeed among the OTUs that best correlated with peak ETM. Sequences related to Desulfobacteraceae and Micrococcineae have also previously been found to be enriched in Columbia River ETM metagenomes (Smith et al., 2013). The former are indirectly involved in organic matter remineralization as they utilize H_2_ and acetate produced during the final steps this process, while Micrococcineae are soil bacteria contributing to the decomposition of organic matter (Smith et al., 2013). Bacteroidetes have been found to be directly involved in water column particle colonization and phytoplankton bloom degradation in this estuary (Smith et al., 2017). These data, together with those of previous studies (Crump et al., 1999; Smith et al., 2013), demonstrate that the difference between ETM particle‐attached and free‐living bacterial communities is a conserved feature of the Columbia River ETM regardless of location, year or season. Furthermore, the fact that all brackish free‐living samples were similar regardless of their origin (ETM or non‐ETM) indicates that the difference in bacterial composition between ETM and non‐ETM waters resided within the community established in their particles. This suggests that brackish water type (ETM or non‐ETM) is a major factor impacting particle‐attached bacteria communities in the CRE and highlights the importance of ETM as retentive areas in fast‐flowing systems, especially for organic matter remineralization. Therefore, our data should be considered in light of the inconsistent findings found in the coastal margins literature with both phylogenetic differences (DeLong et al., 1993; Acinas et al., 1999; Crump et al., 1999; Moesender et al., 2001; Ghiglione et al., 2009; Smith et al., 2013; Jackson et al., 2014; among others) and similarities (Ghiglione et al., 2007; Hollibaugh et al., 2000; Riemann & Winding, 2001) between communities of free‐living and particle‐attached bacteria reported. Importantly, our data demonstrate that the sampled brackish water type (ETM vs. non‐ETM) is an important factor to consider in explaining these conflicting results in estuarine studies.

When putting aside the bacterial community composition data, and instead considering microbial heterotrophic production alone, the distinction between ETM and non‐ETM waters is, at first glance, less clear. Past studies have shown that (1) 90% of the microbial heterotrophic production was associated with particle‐attached bacteria in both ETM and less turbid waters, and (2) the highest rates were measured both in waters collected higher in the water column at the peak of the ETM and in bottom waters slightly before the peak ETM (Crump & Baross, 1996, 2000). These authors hypothesized this pattern to be the result of macroaggragate disaggregation (>3 μm; Reed & Donovan, 1994) ahead of the turbidity peak. Our data confirm their findings, since ~80% of the bulk heterotrophic microbial production was associated with particle‐attached bacteria in samples impacted by an ETM event, but not in pre/post‐ETM event waters that are not impacted by a turbidity event (Tables 1 and 3). It is important to note that the term “ETM” denotes the bottom water mass with peak turbidity (highest SPM concentration) during an ETM event, while “ETM‐impacted” refers here to water present throughout the water column (surface to bottom) during the entire ETM event (from initial rise of turbidity to decline of turbidity). It is also important to point out that our rates (0.13–4.76 mg C L^−1^ hr^−1^, Table 3) are on par with those found in other estuaries. For example, Goosen et al. (1999) reports 0.8–4.5, 2–16 and 0.1–2.8 mg C L^−1^ hr^−1^ for the Elbe, West and Gironde estuaries, respectively. Servais and Garnier (2006) reports 0.15–1.7 and 0.3–2.5 mg C L^−1^ hr^−1^ for the free‐living and particle‐attached bacteria of the Seine estuary. Thus, our data contribute to the body of evidence presented in the coastal margin literature whereby the bulk of heterotrophic production has often been found to be associated with particle‐attached bacteria rather than with their free‐living counterparts (Campbell & Kirchman, 2013; Crump & Baross, 1996; Ghiglione et al., 2007; Kirchman, 1993), although this has not always been observed (Karrascha et al., 2003; Painchaud & Therriault, 1989; Plummer et al., 1987). Clearly, in estuarine studies it is becoming increasingly important to consider whether or not the sampled brackish water mass is impacted by an ETM event when comparing bulk heterotrophic production of particle‐attached and free‐living bacteria.

Although we did not directly characterize particles, we collected different brackish water masses during our time‐series analyses of ETM events: ETM, ETM‐impacted, not ETM‐impacted. The fact that particle‐attached bacterial community composition and bulk heterotrophic production show consistent patterns of variation between these brackish water masses points to the existence of three types of bacteria‐containing particles. The first type populates the ETM (peak turbidity) and hosts a bacterial assemblage characterized by high bulk heterotrophic production. This bacterial assemblage is different from those detected in other estuarine brackish waters, but similar to those of other ETM that are subjected to the same physical forcing. The second particle type is found in waters impacted by an ETM event and are characterized by a high bulk microbial heterotrophic production and a bacterial community that is similar to that of brackish free‐living bacteria. The third type comprises particles of brackish waters that are not impacted by ETM. Those particles have a low bulk microbial heterotrophic production and do not host a distinct bacterial community.

Our study highlights some of the dynamics of the CRE bacterial assemblages, but five fully characterized ETM events cannot capture the entire estuarine complexity. Future studies will need to include other seasons and physical regimes, so that ultimately metrics that define the microbial complexity can be applied to the estuarine biogeochemical models. Recent technological advances enabling autonomous high‐resolution and adaptive sampling of free‐living and particle‐attached bacteria (Herfort et al., 2016) coupled with comparative metagenomic and metatranscriptomic analyses will be instrumental in further characterizing the complex estuarine microbial processes operating within the bacterial assemblages uncovered in this study.

## References

[mbo3522-bib-0001] Acinas, S. G. , Antón, J. , & Rodríguez‐Valera, F. (1999). Diversity of free‐living and attached bacteria in offshore Western Mediterranean waters as depicted by analysis of genes encoding 16S rRNA. Applied and Environmental Microbiology, 65, 514–522. 9925576 10.1128/aem.65.2.514-522.1999 PMC91055

[mbo3522-bib-0002] Almeida, M. A. , Cunha, M. A. , & Alcântara, F. (2002). Seasonal change in the proportion of bacterial and phytoplankton production along a salinity gradient in a shallow estuary. Hydrobiologia, 475(476), 251–262.

[mbo3522-bib-0003] Baptista, A. M. , Seaton, C. , Wilkin, M. , Riseman, S. , Needoba, J. A. , Maier, D. , … Simon, H. M. (2015). Infrastructure for collaborative science and societal applications in the CRE. Frontiers of Earth Science, 9, 659–682. https://doi.org/10.1007/s11707-015-0540-5

[mbo3522-bib-0004] Bouvier, T. C. , & del Giorgio, P. A. (2002). Compositional changes in free‐living bacterial communities along a salinity gradient in two temperate estuaries. Limnology and Oceanography, 47, 453–470.

[mbo3522-bib-0005] Campbell, B. J. , & Kirchman, D. L. (2013). Bacterial diversity, community structure and potential growth rates along an estuarine salinity gradient. International Society for Microbial Ecology Journal, 7, 210–220. https://doi.org/10.1038/ismej.2012.93 10.1038/ismej.2012.93 PMC3526181 22895159

[mbo3522-bib-0006] Clarke, K. R. , Somerfield, P. J. , & Gorley, R. N. (2008). Testing null hypotheses in exploratory community analyses: Similarity profiles and biota‐environmental linkage. Journal of Experimental Marine Biology and Ecology, 366, 56–69. https://doi.org/10.1016/j.jembe.2008.07.009

[mbo3522-bib-0007] Crump, B. C. , Amaral‐Zettler, L. A. , & Kling, G. W. (2012). Microbial diversity in arctic freshwaters is structured by inoculation of microbes from soils. International Society for Microbial Ecology Journal, 6, 1629–1639. https://doi.org/10.1038/ismej.2012.9 10.1038/ismej.2012.9 PMC3498914 22378536

[mbo3522-bib-0008] Crump, B. C. , Armbrust, E. V. , & Baross, J. A. (1999). Phylogenetic analysis of particle‐attached and free‐living bacterial communities in the Columbia River, its estuary and the adjacent coastal ocean. Applied and Environmental Microbiology, 65, 3192–3204. 10388721 10.1128/aem.65.7.3192-3204.1999 PMC91474

[mbo3522-bib-0009] Crump, B. C. , & Baross, J. A. (1996). Particle‐attached bacteria and heterotrophic plankton associated with the CRE turbidity maxima. Marine Ecology Progress Series, 138, 265–273.

[mbo3522-bib-0010] Crump, B. C. , & Baross, J. A. (2000). Characterization of the bacterially‐active particle fraction in the CRE, USA. Marine Ecology Progress Series, 206, 13–22.

[mbo3522-bib-0011] Crump, B. C. , Baross, J. A. , & Simenstad, C. A. (1998). Dominance of particle‐attached bacteria in the CRE, USA. Aquatic Microbial Ecology, 14, 7–18.

[mbo3522-bib-0012] DeLong, E. F. , Franks, D. G. , & Alldredge, A. L. (1993). Phylogenetic diversity of aggregate‐attached vs. free‐living marine bacterial assemblages. Limnology and Oceanography, 38, 924–934.

[mbo3522-bib-0013] Dupont, C. L. , Larsson, J. , Yooseph, S. , Ininbergs, K. , Goll, J. , Asplund‐Samuelsson, J. , … Bergman, B. (2014). Functional tradeoffs underpin salinity‐driven divergence in microbial community composition. PLoS ONE, 9(2), e89549 https://doi.org/10.1371/journal.pone.0089549 24586863 10.1371/journal.pone.0089549 PMC3937345

[mbo3522-bib-0014] Fain, A. M. V. , Jay, D. A. , Wilson, D. J. , Orton, P. M. , & Baptista, A. M. (2001). Seasonal and tidal monthly patterns of particulate dynamics in the Columbia River estuary. Estuaries, 24, 770–786.

[mbo3522-bib-0015] Fortunato, C. S. , & Crump, B. C. (2011). Bacterioplankton community variation across river to ocean environmental gradients. Microbial Ecology, 62, 374–382. https://doi.org/10.1007/s00248-011-9805-z 21286702 10.1007/s00248-011-9805-z

[mbo3522-bib-0016] Fortunato, C. S. , & Crump, B. C. (2015). Microbial gene abundance and expression patterns across a river to ocean salinity gradient. PLoS ONE, 10, e0140578 https://doi.org/10.1371/journal.pone.0140578 26536246 10.1371/journal.pone.0140578 PMC4633275

[mbo3522-bib-0017] Fortunato, C. S. , Eiler, A. , Herfort, L. , Needoba, J. A. , Peterson, T. D. , & Crump, B. C. (2013). Determining indicator taxa across spatial and seasonal gradients in the Columbia River coastal margin. International Society for Microbial Ecology Journal, 7, 1899–1911. https://doi.org/10.1038/ismej.2013.79 10.1038/ismej.2013.79 PMC3965310 23719153

[mbo3522-bib-0018] Fortunato, C. S. , Herfort, L. , Zuber, P. , Baptista, A. M. , & Crump, B. C. (2012). Spatial variability overwhelms seasonal patterns in bacterioplankton communities across a river to ocean gradient. International Society for Microbial Ecology Journal, 6, 554–563. https://doi.org/10.1038/ismej.2011.135 10.1038/ismej.2011.135 PMC3280145 22011718

[mbo3522-bib-0020] Gattuso, J.‐P. , Frankignoulle, M. , & Wollast, R. (1998). Carbon and carbonate metabolism in coastal aquatic ecosystems. Annual Review of Ecology and Systematics, 29, 405–434. https://doi.org/10.1146/annurev.ecolsys.29.1.405

[mbo3522-bib-0021] Geyer, W. R. , & MacCready, P. (2014). The estuarine circulation. Annual Review of Fluid Mechanics, 46, 175–197. https://doi.org/10.1146/annurev-fluid-010313-141302

[mbo3522-bib-0022] Ghiglione, J.‐F. , Conan, P. , & Pujo‐Pay, M. (2009). Diversity of total and active free‐living vs. particle‐attached bacteria in the euphotic zone of the NW Mediterranean Sea. Federation of European Microbiological Societies Microbiology Letters, 299, 9–21. https://doi.org/10.1111/j.1574-6968.2009.01694.x 10.1111/j.1574-6968.2009.01694.x 19686348

[mbo3522-bib-0023] Ghiglione, J. F. , Mével, G. , Pujo‐Pay, M. , Mousseau, L. , Lebaron, P. , & Goutx, M. (2007). Diel and seasonal variations in abundance, activity, and community structure of particle‐attached and free‐living bacteria in NW Mediterranean Sea. Microbial Ecology, 54, 217–231. https://doi.org/10.1007/s00248-006-9189-7 17345139 10.1007/s00248-006-9189-7

[mbo3522-bib-0024] Goosen, N. K. , Kromkamp, J. , Peene, J. , van Rijswijk, P. , & van Breugel, P. (1999). Bacterial and phytoplankton production in the maximum turbidity zone of three European estuaries: The Elbe, Westerschelde and Gironde. Journal of Marine Systems, 22, 151–171.

[mbo3522-bib-0025] Gower, J. C. (1975). Generalized procrustes analysis. Psychometrika, 40, 33–51.

[mbo3522-bib-0026] Hamady, M. , Walker, J. J. , Harris, J. K. , Gold, N. J. , & Knight, R. (2008). Error‐correcting barcoded primers for pyrosequencing hundreds of samples in multiplex. Nature Methods, 5, 235–237. https://doi.org/10.1038/nmeth.1184 18264105 10.1038/nmeth.1184 PMC3439997

[mbo3522-bib-0027] Herfort, L. , Peterson, T. D. , McCue, L. A. , & Zuber, P. (2011). Protist 18S rRNA gene sequence analysis reveals multiple sources of organic matter contributing to turbidity maxima of the CRE. Marine Ecology Progress Series, 438, 19–31. https://doi.org/10.3354/meps09303

[mbo3522-bib-0028] Herfort, L. , Peterson, T. D. , Prahl, F. G. , McCue, L. A. , Needoba, J. A. , Crump, B. C. , … Zuber, P. (2012). Red waters of *Myrionecta rubra* are biogeochemical hotspots for the CRE with impacts on primary/secondary productions and nutrient cycles. Estuaries and Coasts, 35, 878–891. https://doi.org/10.1007/s12237-012-9485-z

[mbo3522-bib-0029] Herfort, L. , Seaton, C. , Wilkin, M. , Roman, B. , Preston, C. M. , Marin, R. III , … Simon, H. M. (2016). Use of continuous, real‐time observations and model simulations to achieve autonomous, adaptive sampling of microbial processes with a robotic sampler. Limnology and Oceanography Methods, 14, 50–67. https://doi.org/10.1002/lom3.10069

[mbo3522-bib-0030] Herlemann, D. P. R. , Labrenz, M. , Jürgens, K. , Bertilsson, S. , Waniek, J. J. , & Andersson, A. F. (2011). Transitions in bacterial communities along the 2000 km salinity gradient of the Baltic Sea. International Society for Microbial Ecology Journal, 5, 1571–1579. https://doi.org/10.1038/ismej.2011.41 10.1038/ismej.2011.41 PMC3176514 21472016

[mbo3522-bib-0031] Herlemann, D. P. R. , Lundin, D. , Andersson, A. F. , Labrenz, M. , & Jürgens, K. (2016). Phylogenetic signals of salinity and season in bacterial community composition across the salinity gradient of the Baltic Sea. Frontiers in Microbiology, 7, 1883 https://doi.org/10.3389/fmicb.2016.01883 27933046 10.3389/fmicb.2016.01883 PMC5121245

[mbo3522-bib-0032] Herman, P. M. J. , & Heip, C. H. R. (1999). Biogeochemistry of the MAximum TURbidity Zone of Estuaries (MATURE): Some conclusions. Journal of Marine Systems, 22, 89–104.

[mbo3522-bib-0033] Hollibaugh, J. T. , Wong, P. S. , & Murrell, M. C. (2000). Similarity of particle‐associated and free‐living bacterial communities in northern San Francisco Bay, California. Aquatic Microbial Ecology, 21, 103–114.

[mbo3522-bib-0034] Hugerth, L. W. , Larsson, J. , Alneberg, J. , Lindh, M. V. , Legrand, C. , Pinhassi, J. , & Andersson, A. F. (2015). Metagenome‐assembled genomes uncover a global brackish microbiome. Genome Biology, 16, 279 https://doi.org/10.1186/s13059-015-0834-7 26667648 10.1186/s13059-015-0834-7 PMC4699468

[mbo3522-bib-0035] Jackson, D. (1995). PROTEST: A PROcrustean Randomization TEST of community environment concordance. Ecoscience, 2, 297–303.

[mbo3522-bib-0036] Jackson, C. R. , Millar, J. J. , Payne, J. T. , & Ochs, C. A. (2014). Free‐living and particle‐associated bacterioplankton in large rivers of the Mississippi river basin demonstrate biogeographic patterns. Applied and Environmental Microbiology, 80, 7186–7195. https://doi.org/10.1128/AEM.01844-14 25217018 10.1128/AEM.01844-14 PMC4249191

[mbo3522-bib-0037] Jay, D. A. , & Musiak, J. D. (1996). Internal tidal asymmetry in Channel flows: Origins and consequences In Pattiaratchi C. (Ed.), Mixing processes in estuaries and coastal seas (pp. 219–258). Washington, D.C: American Geophysical Union Coastal and Estuarine Sciences Monograph.

[mbo3522-bib-0038] Jay, D. A. , & Smith, J. D. (1990). Residual circulation in shallow estuaries: 1. Highly stratified, narrow estuaries. Journal Geophysical Research: Oceans, 95, 711–731.

[mbo3522-bib-0039] Karrascha, B. , Ullrichb, S. , Mehrensa, M. , & Zimmermann‐Timm, H. (2003). Free and particle‐associated extracellular enzyme activity and bacterial production in the lower Elbe estuary, Germany. Acta Hydrochimica et Hydrobiologica, 31, 297–306. https://doi.org/10.1002/aheh.200300505

[mbo3522-bib-0040] Kirchman, D. L. (1993). Particulate detritus and bacteria in marine environments In Ford T. (Ed.), Aquatic microbiology: An ecological approach (pp. 321–341). Cambridge, MA: Blackwell.

[mbo3522-bib-0041] Kirchman, D. L. , Dittel, A. I. , Malmstrom, R. R. , & Cottrell, M. T. (2005). Biogeography of major bacterial groups in the Delaware estuary. Limnology and Oceanography, 50, 1697–1706.

[mbo3522-bib-0042] Kirchman, D. L. , Keil, R. G. , Simon, M. , & Welschmeyer, N. A. (1993). Biomass and production of heterotrophic bacterioplankton in the oceanic subarctic Pacific. Deep‐Sea Research, 40, 967–988.

[mbo3522-bib-0043] Lane, D. J. (1991). 16S/23S rRNA sequencing In Stackebrandt E., & Goodfellow M. (Eds.), Nucleic acid techniques in bacterial systematic (pp. 115–175). New York: John Wiley and Sons press.

[mbo3522-bib-0044] Lapoussière, A. , Michel, C. , Starr, M. , Gosselin, M. , & Poulin, M. (2011). Role of free‐living and particle‐attached bacteria in the recycling and export of organic material in the Hudson Bay system. Journal of Marine Systems, 88, 434–445. https://doi.org/10.1016/j.jmarsys.2010.12.003

[mbo3522-bib-0045] Lara‐Lara, J. R. , Frey, B. E. , & Small, L. F. (1990). Primary production in the CRE. I. spatial and temporal variability of properties. Pacific Science, 44, 17–37.

[mbo3522-bib-0046] Liu, J. , Fu, B. , Yang, H. , Zhao, M. , He, B. , & Zhang, X.‐H. (2015). Phylogenetic shifts of bacterioplankton community composition along the Pearl estuary: The potential impact of hypoxia and nutrients. Frontiers in Microbiology, 6 article 64. https://doi.org/10.3389/fmicb.2015.00064 10.3389/fmicb.2015.00064 PMC4322608 25713564

[mbo3522-bib-0047] Lopez, J. , Baptista, A. M. , & Spitz, Y . (2012). Modeling estuarine turbidity maxima in the Columbia River estuary 2012 Columbia River estuary conference: New scientific findings and their management implications, Astoria, Oregon, USA.

[mbo3522-bib-0049] Maher, D. T. , & Eyre, B. D. (2012). Carbon budgets for three autotrophic Australian estuaries: Implications for global estimates of the coastal air‐water CO_2_ flux. Global Biogeochemical Cycles, 26, GB1032 https://doi.org/10.1029/2011gb004075

[mbo3522-bib-0050] Moesender, M. M. , Winter, C. , & Herndl, G. J. (2001). Horizontal and vertical complexity of attached and free‐living bacteria of the eastern Mediterranean Sea, determined by 16S rDNA and 16S rRNA fingerprints. Limnology and Oceanography, 46, 95–107. https://doi.org/10.4319/lo.2001.46.1.0095

[mbo3522-bib-0052] Ortega‐Retuerta, E. , Joux, F. , Jeffrey, W. H. , & Ghiglione, J. F. (2013). Spatial variability of particle‐attached and free‐living bacterial diversity in surface waters from the Mackenzie River to the Beaufort Sea (Canadian Arctic). Biogeosciences, 10, 2747–2759. https://doi.org/10.5194/bg-10-2747-2013

[mbo3522-bib-0053] Painchaud, J. , & Therriault, J. C. (1989). Relationships between bacteria, phytoplankton and particulate organic carbon in the Upper St Lawrence estuary. Marine Ecology Progress Series, 56, 301–311.

[mbo3522-bib-0054] Plummer, D. H. , Owens, N. J. P. , & Herbert, R. A. (1987). Bacteria‐particle interactions in turbid estuarine environments. Continental Shelf Research, 7, 1429–1433.

[mbo3522-bib-0055] Prahl, F. G. , Small, L. F. , & Eversmeyer, B. (1997). Biogeochemical characterization of suspended particulate matter in the CRE. Marine Ecology Progress Series, 160, 173–184.

[mbo3522-bib-0056] Reed, D. J. , & Donovan, J . (1994). Character and composition of the Columbia River estuarine turbidity maximum In Dyer K. & Orth R. (Eds.), Changing particle fluxes in estuaries: Implications from science to management (pp. 445–450). ECSAERF22 Symposium, Plymouth, September 1992, Friedensborg: Olsen & Olsen Press.

[mbo3522-bib-0057] Rieck, A. , Herlemann, D. P. R. , Jürgens, K. , & Grossart, H.‐P. (2015). Particle‐associated differ from free‐living bacteria in surface waters of the Baltic Sea. Frontiers in Microbiology, 6, 1297 https://doi.org/10.3389/fmicb.2015.01297 26648911 10.3389/fmicb.2015.01297 PMC4664634

[mbo3522-bib-0058] Riemann, L. , & Winding, A. (2001). Community dynamics of free‐living and particle‐associated bacterial assemblages during a freshwater phytoplankton bloom. Microbial Ecology, 42, 274–285. https://doi.org/10.1007/s00248-001-0018-8 12024253 10.1007/s00248-001-0018-8

[mbo3522-bib-0059] Sanford, L. P. , Suttles, S. E. , & Halka, J. P. (2001). Reconsidering the physics of the Chesapeake Bay estuarine turbidity maximum. Estuaries, 24, 655–669. https://doi.org/10.2307/1352874

[mbo3522-bib-0060] Santegoeds, C. M. , Ferdelman, T. G. , Muyzer, G. , & de Beer, D. (1998). Structural and functional dynamics of sulfate‐reducing populations in bacterial biofilms. Applied and Environmental Microbiology, 64, 3731–3739. 9758792 10.1128/aem.64.10.3731-3739.1998 PMC106533

[mbo3522-bib-0061] Santos, L. , Vaz, L. , Marcial Gomes, N. C. , Vaz, N. , Miguel Dias, J. , Cunha, Â. , & Almeida, A. (2014). Impact of freshwater inflow on bacterial abundance and activity in the estuarine system Ria de Aveiro. Estuarine, Coastal and Shelf Science, 138, 107–120. https://doi.org/10.1016/j.ecss.2013.12.021

[mbo3522-bib-0062] Schloss, P. D. , Westcott, S. L. , Ryabin, T. , Hall, J. R. , Hartmann, M. , Hollister, E. B. , … Weber, C. F. (2009). Introducing mothur: Open‐source, platform‐independent, community‐supported software for describing and comparing microbial communities. Applied and Environmental Microbiology, 75, 7537–7541. https://doi.org/10.1128/AEM.01541-09 19801464 10.1128/AEM.01541-09 PMC2786419

[mbo3522-bib-0164] Servais, P. , & Garnier, J. , (2006). Organic carbon and bacterial heterotrophic activity in the maximum turbidity zone of the Seine estuary (France). Aquatic Science, 68, 78–85.

[mbo3522-bib-0063] Sherwood, C. R. , Creager, J. S. , Roy, E. H. , Gelfenbaum, G. , & Dempsey, T. (1984). Sedimentary processes and environments in the Columbia River Estuary. Astoria, OR: Columbia River Estuary Data Development Program, Columbia River Estuary Study Taskforce.

[mbo3522-bib-0065] Simon, M. , Grossart, H.‐P. , Schweitzer, B. , & Ploug, H. (2002). Microbial ecology of organic aggregates in aquatic ecosystems. Aquatic Microbial Ecology, 28, 175–211. https://doi.org/10.3354/ame028175

[mbo3522-bib-0066] Small, L. F. , McIntire, C. D. , MacDonald, K. B. , Lara‐Lara, J. R. , Frey, B. E. , Amspoker, M. C. , & Winfield, T. (1990). Primary production, plant and detrital biomass, and particle transport in the CRE. Progress in Oceanography, 25, 175–210.

[mbo3522-bib-0067] Small, L. F. , & Morgan, S. R . (1994). Phytoplankton attributes in the turbidity maximum of the CRE, USA In Dyer K. & Orth R. (Eds.), Changing particle fluxes in estuaries: Implications from science to management (pp. 465–472), ECSAERF22 Symposium, Plymouth, September 1992, Friedensborg, Olsen & Olsen Press.

[mbo3522-bib-0068] Small, L. F. , & Prahl, F. G. (2004). A particle conveyor belt process in the CRE: Evidence from chlorophyll a and particulate organic carbon. Estuaries, 27, 999–1013.

[mbo3522-bib-0069] Smith, M. W. , Davis, R. E. , Youngblut, N. D. , Kärnä, T. , Herfort, L. , Whitaker, R. J. , … Simon, H. M. (2015). Metagenomic evidence for reciprocal particle exchange between the mainstem estuary and lateral bay sediments of the lower Columbia River. Frontiers in Microbiology, 6, 1074 https://doi.org/10.3389/fmicb.2015.01074 26483785 10.3389/fmicb.2015.01074 PMC4589670

[mbo3522-bib-0070] Smith, M. W. , Herfort, L. , Fortunato, C. S. , Crump, B. C. , & Simon, H. M. (2017). Microbial players and processes involved in phytoplankton bloom utilization in the water column of a fast‐flowing, river‐dominated estuary. Microbiology Open, e00467. https://doi.org/10.1002/mbo3.467 10.1002/mbo3.467 PMC5552926 28318115

[mbo3522-bib-0071] Smith, M. W. , Herfort, L. , Tyrol, K. , Suciu, D. , Campbell, V. , Crump, B. C. , … Simon, H. M. (2010). Seasonal changes in bacterial and archaeal gene expression patterns across salinity gradients in the Columbia River coastal margin. PLoS ONE, 5, e13312 https://doi.org/10.1371/journal.pone.0013312 20967204 10.1371/journal.pone.0013312 PMC2954162

[mbo3522-bib-0072] Smith, M. W. , Zeigler‐Allen, L. , Allen, A. E. , Herfort, L. , & Simon, H. M. (2013). Contrasting genomic properties of free‐living and particle‐attached microbial assemblages within a coastal ecosystem. Frontiers in Microbiology, 4, 120 https://doi.org/10.3389/fmicb.2013.00120 23750156 10.3389/fmicb.2013.00120 PMC3668451

[mbo3522-bib-0073] Sullivan, B. E. , Prahl, F. G. , Small, L. F. , & Covert, P. A. (2001). Seasonality of phytoplankton production in the Columbia River: A natural or anthropogenic pattern? Geochimica et Cosmochimica Acta, 65, 1125–1139. https://doi.org/10.1016/S0016-7037(00)00565-2

[mbo3522-bib-0074] Wang, Q. , Garrity, G. M. , Tiedje, J. M. , & Cole, J. R. (2007). Naïve Bayesian classifier for rapid assignment of rRNA sequences into the new bacterial taxonomy. Applied Environmental Microbiology, 73, 5261–5267. https://doi.org/10.1128/AEM.00062-07 17586664 10.1128/AEM.00062-07 PMC1950982

[mbo3522-bib-0075] Williams, T. J. , Long, E. , Evans, F. , DeMaere, M. Z. , Lauro, F. M. , Raftery, M. J. , … Cavicchioli, R. (2012). A metaproteomic assessment of winter and summer bacterioplankton from Antarctic Peninsula coastal surface waters. International Society for Microbial Ecology Journal, 6, 1883–1900. https://doi.org/10.1038/ismej.2012.28 10.1038/ismej.2012.28 PMC3446797 22534610

[mbo3522-bib-0076] Zimmermann, H. (1997). The microbial community on aggregates in the Elbe Estuary, Germany. Aquatic Microbial Ecology, 13, 37–46.

