# Supplementary figures and images for: Factors affecting the bacterial community composition and heterotrophic production of Columbia River estuarine turbidity maxima
Source: Microbiologyopen. 2017 Aug 6;6(6):e00522. doi: 10.1002/mbo3.522 (PMC5727365; doi:10.1002/mbo3.522)

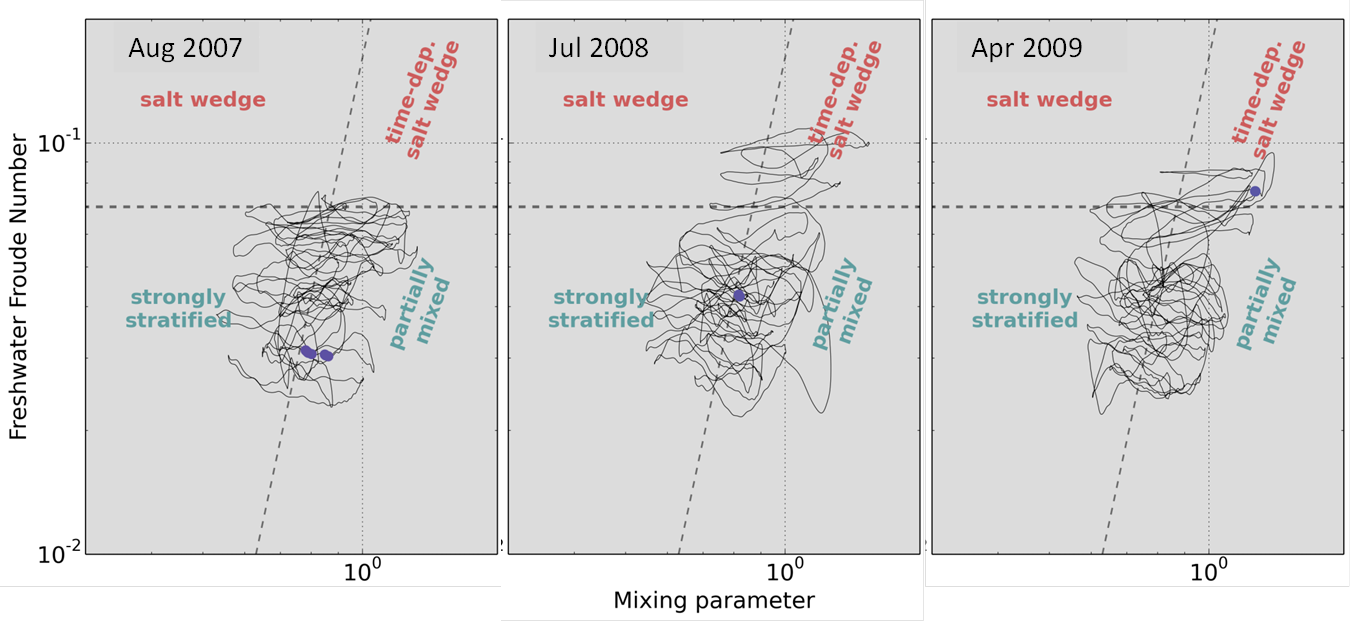

Supplement: Supplementary file 3 [file MBO3-6-na-s003.docx]
