# Supplementary material for: Factors affecting the bacterial community composition and heterotrophic production of Columbia River estuarine turbidity maxima
Source: Microbiologyopen. 2017 Aug 6;6(6):e00522. doi: 10.1002/mbo3.522 (PMC5727365; doi:10.1002/mbo3.522)
Supplement: Supplementary file 12 [file MBO3-6-na-s012.docx]

SUPPLEMENTARY MATERIAL - TABLE S1. Number of sequences and Operational Taxonomic Units (OTU) per sample in our Sanger and pyrosequencing datasets. * = number of OTU when rarefied to 149 sequences; n.a. = not applicable

SUPPLEMENTARY MATERIAL - TABLE S2. Environmental data used to determine best match between environmental data and bacterial community structure. BP = Bacterial production (data from Table 3). Note that BETS-BIOENV analysis can only be done on complete datasets, so when necessary either sample or variable was removed of specific analysis. n.d.= not determined.

SUPPLEMENTARY MATERIAL - TABLE S3. Relative contribution, given as percent of total, of different bacteria 16S rRNA gene sequences to the total number of bacterial clones in libraries constructed from Sanger sequencing of CRE water and sediment samples. Samples collected from the bottom water during the peak of each ETM event are highlighted in bold.

SUPPLEMENTARY MATERIAL - TABLE S4. Biogeochemical indicators of phytoplankton abundance and health (chlorophyll *a* (chl *a*) and particulate organic carbon (POC), and chl *a*/ POC ratio) for a subset of August 2007 and May 2009 water samples. ETM are highlighted in bold.* = data previously published in Herfort et al. 2011

SUPPLEMENTARY MATERIAL - TABLE S5. Relative contribution, given as percent of total, of different bacteria 16S rRNA gene sequences to the total number of bacterial clones in libraries constructed from pyrosequencing of CRE water samples. ETM are highlighted in bold.

SUPPLEMENTARY MATERIAL - Fig. S1. Rarefaction curves of each sample generated for the (A) Sanger sequencing (made in MOTHUR and plotted in Excel) and (B) pyrosequencing (made and plotted in QIIME) datasets.

SUPPLEMENTARY MATERIAL - Fig. S2. Bacterial community composition based on Bray-Curtis similarities of the relative abundances of OTUs identified using the full dataset of pyrosequences (not rarefied). Red = non-significant diversions based on SIMPROF analysis. As for rarefied sequences, ETM samples R & L cluster together while ETM samples K & T do not, and ETM whole and free-living samples do not cluster together, while those of non-ETM do. Also, 2008 samples cluster together, but Ocean samples (L & R) differ enough from ETM samples (K & T) to form their own sub-cluster. Also, eventhough the non-ETM samples of July 2007 still form a distinct cluster, more internal differences are apparent between these samples than in the rarefied dataset. Sample U, which is a surface water sample collected during peak ETM (salinity 6.8 PSU) also does not group with other non-ETM samples in this full dataset. As with the rarefied dataset, we detected a predominance of Bacteroidetes in this full dataset and a relative high abundance of bacteria typically found in oxygen-depleted waters in the Ocean and ETM samples of July 2008 (data not shown).

SUPPLEMENTARY MATERIAL - Fig. S3. Plots of two-parameter space (freshwater Froude number and mixing number) regime of the CRE in 2007-2009. The specific regime of each month studied is indicated by the dots denoting sampling times, while black lines represent the estuarine regime for the whole year. These plots, suggested by Geyer & MacCready (2014) to describe physical behaviors of estuaries, were generated using the simulation data for a transect at the interdisciplinary endurance station SATURN-03 located ~14 km upstream of the entrance of the estuary in the South channel (http://www.stccmop.org).

SUPPLEMENTARY MATERIAL - Fig. S4. Proportion of unique and shared OTU in water (A-P) and sediment (Q) samples of the Sanger sequencing dataset. This shows a high percentage of unique OTU (57%) in the sediment sample (Q).

SUPPLEMENTARY MATERIAL - Fig. S5. Elevation at Astoria (NOAA station 9439040) and North-South wind speed (NOAA National Data Buoy Center station 46029) for August 2007 (A) and July 2008 (B). Data were obtained from the CMOP website (<http://www.stccmop.org/datamart/observation_network/dataexplorer>). Values above and below the dashed line are associated with North and South blowing winds, respectively. A minimum of three continuous days with South blowing winds define an upwelling period (Roegner et al. 2011).

SUPPLEMENTARY MATERIAL - Fig. S6. Percentages of strict and facultative anaerobic bacteria 16S rRNA gene sequences corrected for SPM concentration (= % sequence / [SPM]) for our July 2008 pyrosequencing dataset, plotted against salinity. Dotted line represents a conservative salinity mixing line formulated using the average abundance data of our two marine samples (31.4 PSU for sample L and 31.9 PSU for sample R, Tables 1and 2).
