# Supplementary material for: Factors affecting the bacterial community composition and heterotrophic production of Columbia River estuarine turbidity maxima
Source: Microbiologyopen. 2017 Aug 6;6(6):e00522. doi: 10.1002/mbo3.522 (PMC5727365; doi:10.1002/mbo3.522)
Supplement: Supplementary file 8 [file MBO3-6-na-s001.docx]

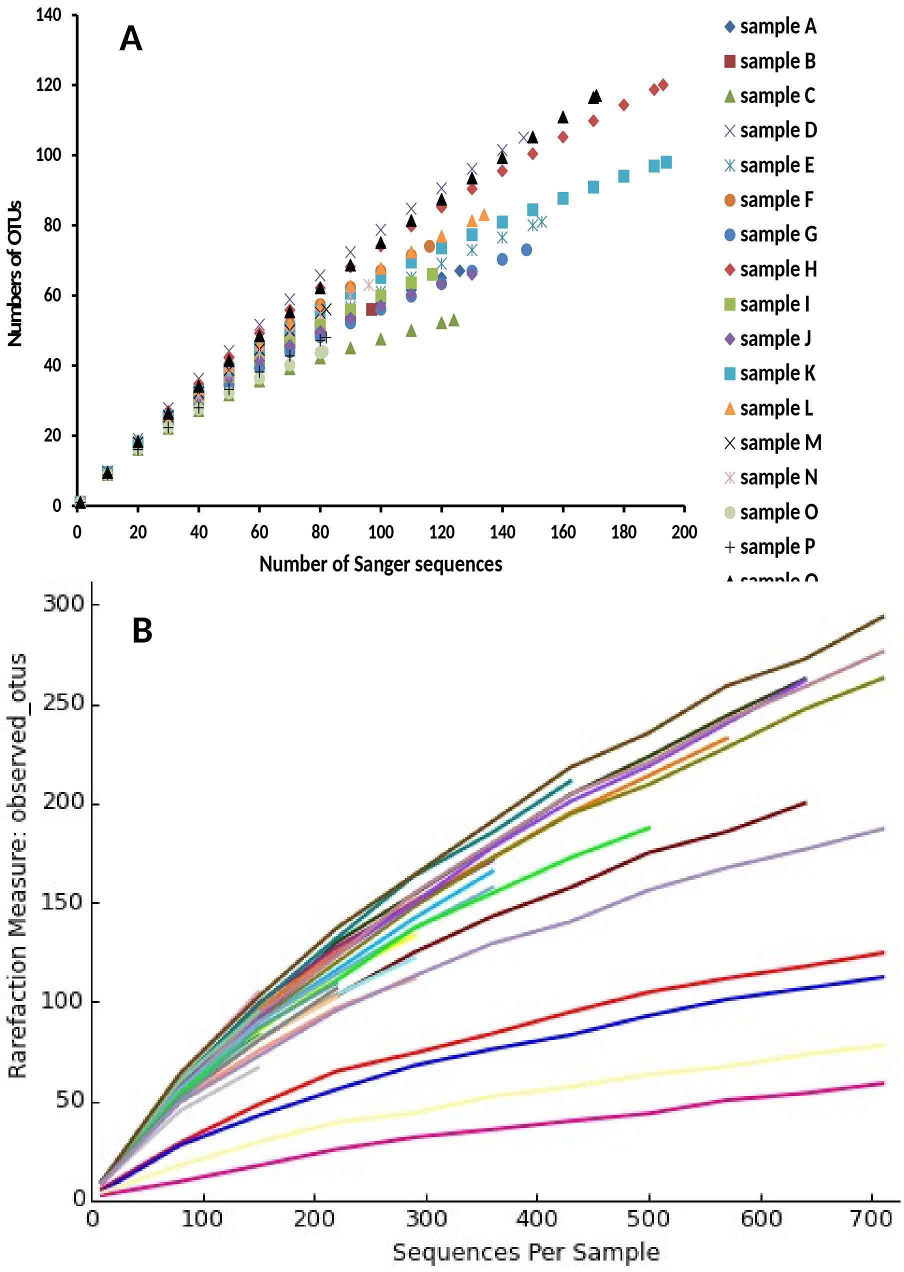
A
Numbers of OTUs
Number of Sanger sequences
sample A
sample B
sample C
sample D
sample E
sample F
sample G
sample H
sample I
sample J
sample K
sample L
sample M
sample N
sample O
sample P
B
Rarefaction Measure: observed_otus
Sequences Per Sample
