# Supplementary material for: Factors affecting the bacterial community composition and heterotrophic production of Columbia River estuarine turbidity maxima
Source: Microbiologyopen. 2017 Aug 6;6(6):e00522. doi: 10.1002/mbo3.522 (PMC5727365; doi:10.1002/mbo3.522)
Supplement: Supplementary file 9 [file MBO3-6-na-s002.docx]

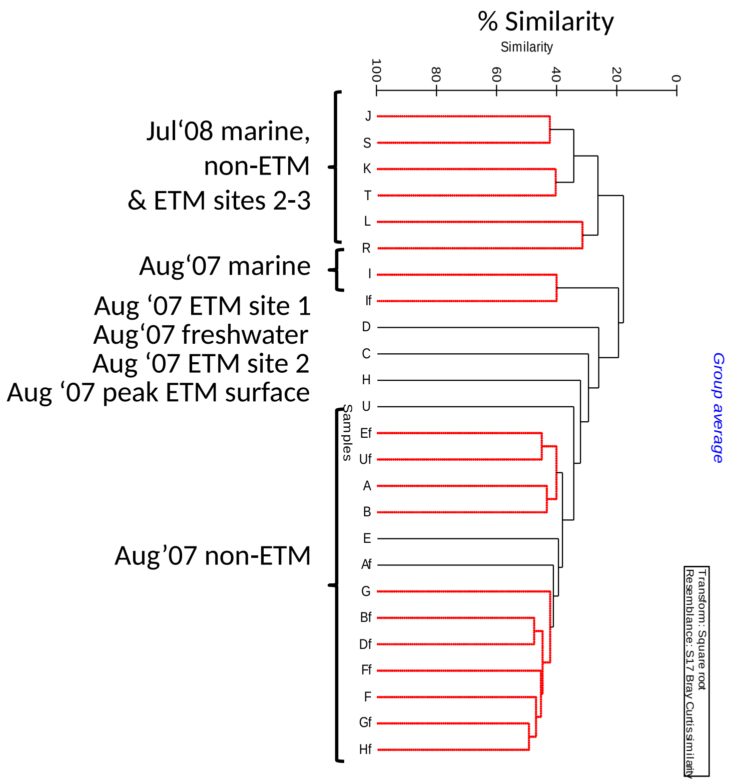
% Similarity
Similarity
100
80
60
40
20
0
Jul'08 marine,
non-ETM
& ETM sites 2-3
Aug'07 marine
Aug '07 ETM site 1
Aug'07 freshwater
Aug '07 ETM site 2
Aug '07 peak ETM surface
Aug'07 non-ETM
Samples
J
S
K
T
L
R
I
If
D
C
H
U
Ef
Uf
A
B
E
Af
G
Bf
Df
Ff
F
Gf
Hf
Group average
Transform: Square root
Resemblance: S17 Bray Curtis similarity
