# Supplementary material for: Factors affecting the bacterial community composition and heterotrophic production of Columbia River estuarine turbidity maxima
Source: Microbiologyopen. 2017 Aug 6;6(6):e00522. doi: 10.1002/mbo3.522 (PMC5727365; doi:10.1002/mbo3.522)
Supplement: Supplementary file 10 [file MBO3-6-na-s004.docx]

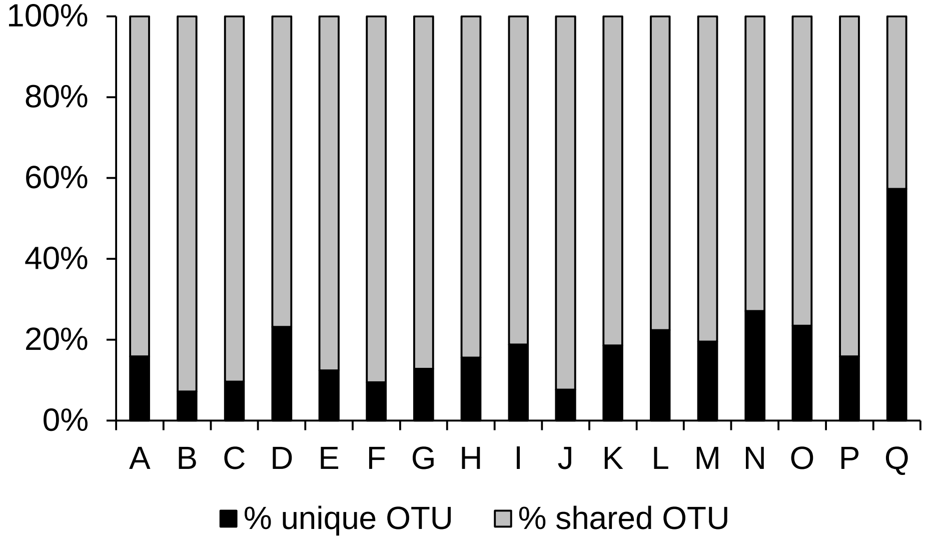
100%
80%
60%
40%
20%
0%
A B C D E F G H I J K L M N O P Q
% unique OTU
% shared OTU
