# Supplementary material for: Factors affecting the bacterial community composition and heterotrophic production of Columbia River estuarine turbidity maxima
Source: Microbiologyopen. 2017 Aug 6;6(6):e00522. doi: 10.1002/mbo3.522 (PMC5727365; doi:10.1002/mbo3.522)
Supplement: Supplementary file 11 [file MBO3-6-na-s005.docx]

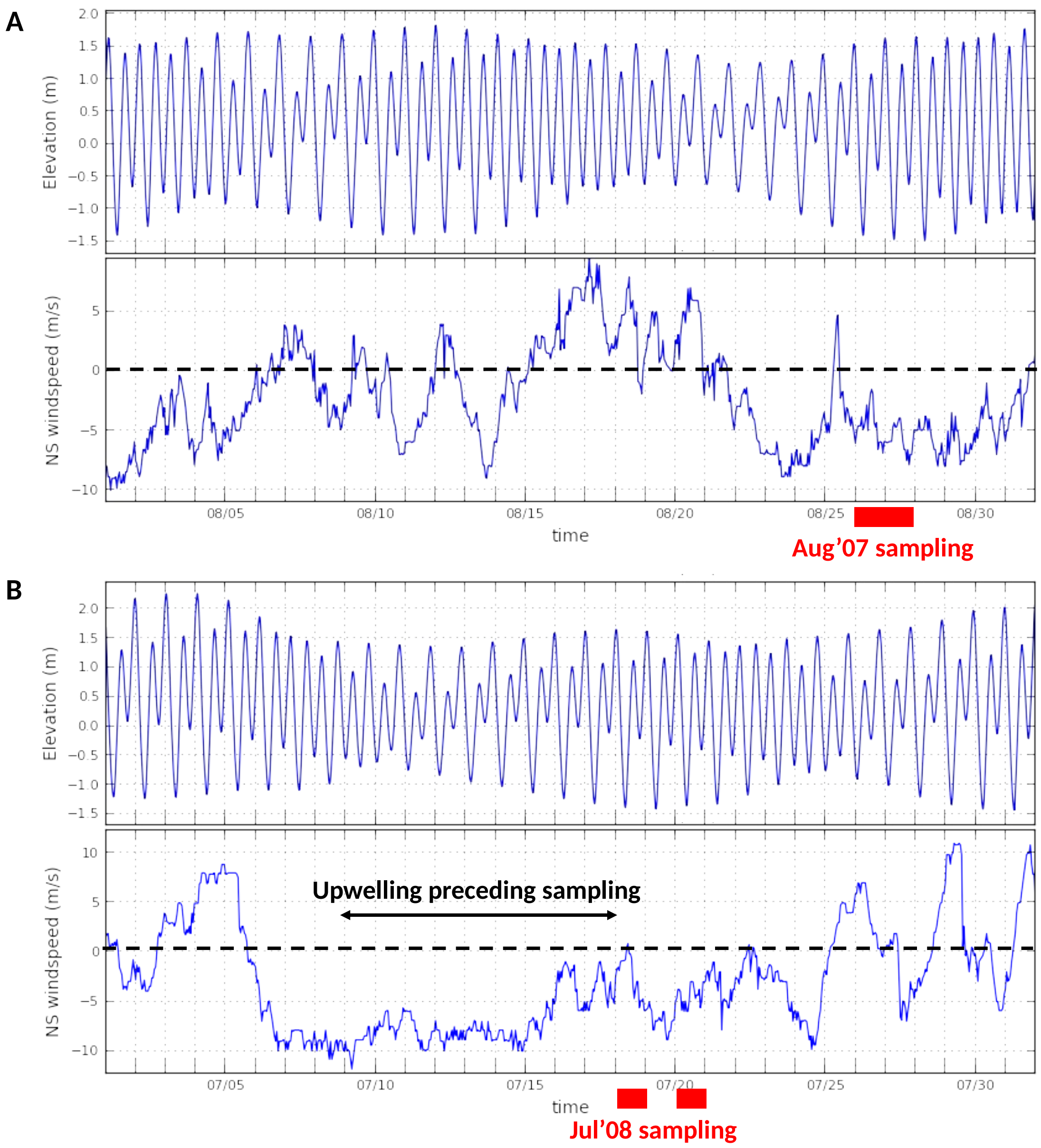
A
Elevation (m)
2.0
1.5
1.0
0.5
0.0
−0.5
−1.0
−1.5
NS windspeed (m/s)
5
0
−5
−10
08/05
08/10
08/15
08/20
08/25
08/30
time
Aug'07 sampling
B
Elevation (m)
2.0
1.5
1.0
0.5
0.0
−0.5
−1.0
−1.5
NS windspeed (m/s)
10
5
0
−5
−10
Upwelling preceding sampling
07/05
07/10
07/15
07/20
07/25
07/30
time
Jul'08 sampling
