# Supplementary material for: Factors affecting the bacterial community composition and heterotrophic production of Columbia River estuarine turbidity maxima
Source: Microbiologyopen. 2017 Aug 6;6(6):e00522. doi: 10.1002/mbo3.522 (PMC5727365; doi:10.1002/mbo3.522)
Supplement: Supplementary file 12 [file MBO3-6-na-s006.docx]

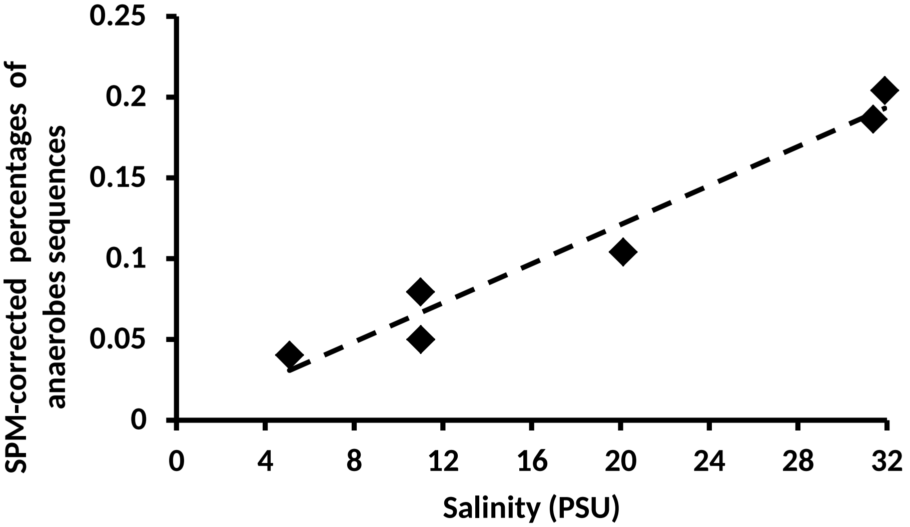
SPM-corrected percentages of anaerobes sequences
0.25
0.2
0.15
0.1
0.05
0
0
4
8
12
16
20
24
28
32
Salinity (PSU)
